# mTORC1 induces plasma membrane depolarization and promotes preosteoblast senescence by regulating the sodium channel Scn1a

**DOI:** 10.1038/s41413-022-00204-1

**Published:** 2022-03-08

**Authors:** Ajuan Chen, Jian Jin, Shasha Cheng, Zezheng Liu, Cheng Yang, Qingjing Chen, Wenquan Liang, Kai Li, Dawei Kang, Zhicong Ouyang, Chenfeng Yao, Xiaochun Bai, Qingchu Li, Dadi Jin, Bin Huang

**Affiliations:** 1 Academy of Orthopedics, Guangdong Province, Guangdong Provincial Key Laboratory of Bone and Joint Degeneration Diseases, Department of Spine Surgery, The Third Affiliated Hospital of Southern Medical University, Guangzhou, China; 2 Department of Spine Surgery, Nanfang Hospital, Southern Medical University, Guangzhou, China; 3 Department of Clinical Laboratory, The Third Affiliated Hospital of Southern Medical University, Guangzhou, China; 4 Department of Cell Biology, School of Basic Medical Science, Southern Medical University, Guangzhou, China

**Keywords:** Osteoporosis, Bone

## Abstract

Senescence impairs preosteoblast expansion and differentiation into functional osteoblasts, blunts their responses to bone formation-stimulating factors and stimulates their secretion of osteoclast-activating factors. Due to these adverse effects, preosteoblast senescence is a crucial target for the treatment of age-related bone loss; however, the underlying mechanism remains unclear. We found that mTORC1 accelerated preosteoblast senescence in vitro and in a mouse model. Mechanistically, mTORC1 induced a change in the membrane potential from polarization to depolarization, thus promoting cell senescence by increasing Ca^2+^ influx and activating downstream NFAT/ATF3/p53 signaling. We further identified the sodium channel Scn1a as a mediator of membrane depolarization in senescent preosteoblasts. Scn1a expression was found to be positively regulated by mTORC1 upstream of C/EBPα, whereas its permeability to Na^+^ was found to be gated by protein kinase A (PKA)-induced phosphorylation. Prosenescent stresses increased the permeability of Scn1a to Na^+^ by suppressing PKA activity and induced depolarization in preosteoblasts. Together, our findings identify a novel pathway involving mTORC1, Scn1a expression and gating, plasma membrane depolarization, increased Ca^2+^ influx and NFAT/ATF3/p53 signaling in the regulation of preosteoblast senescence. Pharmaceutical studies of the related pathways and agents might lead to novel potential treatments for age-related bone loss.

## Introduction

Bone remodeling is mediated mainly by bone-resorbing osteoclasts and bone-forming osteoblasts and their precursors. In the first three decades of healthy human life, bone formation dominates until peak bone mass is achieved.^1^ With aging, bone remodeling is interrupted in favor of bone resorption, which causes bone loss and predisposes the skeleton to fractures. Recent studies have revealed a causal role of senescent cells in the formation-resorption shift in bone remodeling and age-related bone loss. Multiple cell types in the bone microenvironment become senescent with aging.^2^ Eliminating senescent cells, however, has both antiresorptive and anabolic effects on bone.^3^ Moreover, the pathophysiology of senile osteoporosis in aged humans is characterized by a decrease in bone formation that exceeds the decrease in bone resorption.^4^ Therefore, senescence-related osteoblast dysfunction is the main cause of age-related bone loss.^5^

Bone formation requires an adequate number of mature osteoblastic cells, which are postmitotic cells that have a short lifespan and must be continually replaced with new cells.^6^ Osteoblasts arise from preosteoblasts, which express the transcription factors Osterix (Osx) and Runx2.^7^ However, senescence impairs preosteoblast proliferation and differentiation into mature osteoblasts.^8^ Senescent preosteoblasts also exhibit a blunted response to growth factors and hormones known to control bone formation.^9–11^ Moreover, these cells have been reported to create a defective microenvironment for bone formation by secreting several inflammatory and osteoclast-activating factors.^12^ Due to these adverse effects, preosteoblast senescence is a crucial target for the treatment of age-related bone loss; however, the underlying mechanism remains unclear.

As the core signaling pathway in regulating lifespan, mTORC1 is associated with various processes related to aging, such as nutrient sensing, maintenance of proteostasis, autophagy, mitochondrial dysfunction and cellular senescence.^13^ Interestingly, emerging evidence suggests different requirements for mTORC1 in early life vs. late life. mTORC1 is needed for development and reproduction because its functional absence is lethal during embryogenesis.^14^ Later in life, however, active mTORC1 drives senescence and increases the risk of disease.^15^ We previously reported essential roles of mTORC1 in preosteoblast expansion and function during bone development;^16,17^ however, its roles in preosteoblast senescence and bone aging warrant further investigation.

Cells have a transmembrane potential,^18^ which is maintained by the balance between ions on both sides of the plasma membrane. Although the underlying mechanisms are unclear, membrane potential changes have been revealed to be involved in the senescence of fibroblasts^19^ and epithelial cells.^20^ Because mTORC1 has been reported to be associated with membrane depolarization, we investigated whether membrane depolarization has a role in mTORC1-regulated preosteoblast senescence and further unveiled the underlying mechanisms. Hence, the present study sheds new light on a novel electrophysiological pathway controlling preosteoblast senescence and bone aging.

## Results

### mTORC1 is activated in senescent preosteoblasts

**Fig. 1:**
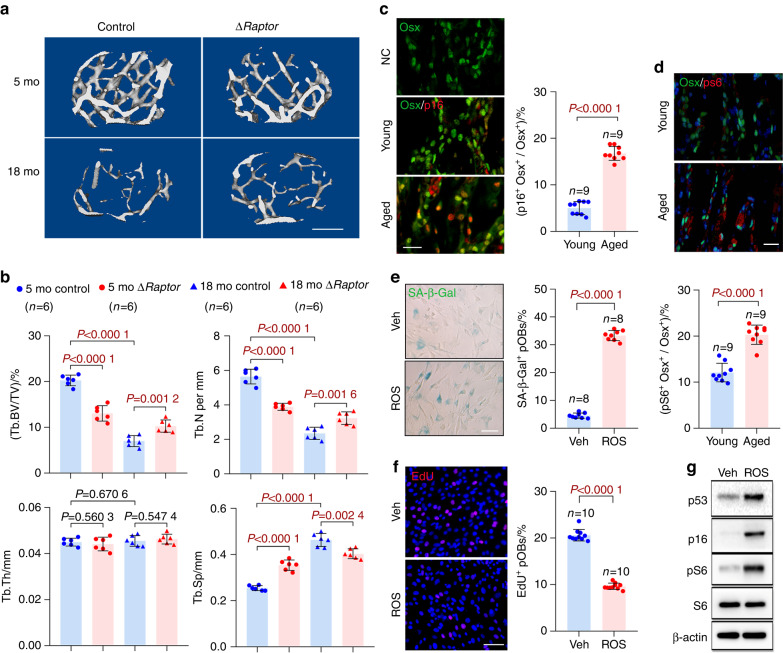
Senescent preosteoblasts present elevated mTORC1 activity. **a** Representative microcomputed tomography (μCT) images of femurs of 5-month-old (mo) and 18-month-old (mo) Δ*Raptor* mice compared with those of the littermate controls. Scale bar: 500 μm. **b** Quantification of trabecular bone volume per total volume (Tb.BV/TV), trabecular number (Tb.N), trabecular separation (Tb.Sp) and trabecular thickness (Tb.Th). Representative images of double immunostaining of Osx plus p16 (**c**) and Osx plus pS6 (Ser235/236) (**d**) in the bones of the mice in **a**. Single Osx staining served as a negative control (NC) for double staining of Osx plus p16 in **c**. Scale bars: 100 μm. **e** The murine preosteoblast cell line MC3T3-E1 was exposed to senescence induction by reactive oxygen species (ROS) or treated with vehicle (Veh) and stained for the expression of senescence-associated β-galactosidase (SA-β-gal) 3 days later. Scale bar: 50 μm. Quantification of the proportion of SA-β-gal^+^ preosteoblasts (pOBs) in each population is shown. **f** Representative confocal images of immunostaining of EdU (red) in the cells in **e** and quantitative analysis of EdU^+^ pOBs relative to total cells (%). Scale bar, 100 μm. **g** Western blot analysis of senescence marker (p16 and p53) expression and mTORC1 activity [pS6(Ser235/236)] in the cells in **e**. Data are shown as the mean ± SD. The numbers of samples (*n*) are indicated in each figure panel. *P* values were determined by two-tailed Student’s *t* test for single comparisons

We^17^ and other groups^21,22^ have reported that mTORC1 inactivation by deletion of *Raptor* (mTORC1-specific component) in preosteoblasts (Δ*Raptor*) causes lower bone mass in young mice. Interestingly, these mice regained bone mass to an even greater extent than the littermate controls at an old age (18 months) (Fig. 1a, b), thus indicating the different roles of mTORC1 in preosteoblasts during bone aging versus development.

To determine the role of mTORC1 during preosteoblast senescence, we first examined mTORC1 levels in preosteoblasts during senescence. As reported previously,^8^ the numbers of both Osx-positive preosteoblasts (Fig. S1a) and their descendants, osteocalcin (Ocn)-positive osteoblasts (Fig. S1b), were lower in the bones of the old mice (18 months) than the young mice (9 months). Importantly, the senescent preosteoblasts in the old mice (Fig. 1c) showed significantly elevated mTORC1 activity, as revealed by the phosphorylation levels of S6 (Ser235/236) (Fig. 1d).

To confirm these results in vitro, we further induced senescence in the MC3T3-E1 preosteoblast cell line by using reactive oxygen species (ROS). ROS treatment increased senescence-associated-β-galactosidase (SA-β-Gal) activity (Fig. 1e) and proliferative arrest (Fig. 1f) and elevated senescence marker (p16 and p53) expression (Fig. 1g) in the cells, all of which are major hallmarks of senescence. As expected, S6 (Ser235/236) phosphorylation was elevated in these senescent cells (Fig. 1g). Primary calvarial osteoblasts showed replicative exhaustion at population doubling level (PDL) 25 (Fig. S2a–e). Greater S6 (Ser235/236) phosphorylation was also observed in these replicative senescent cells than in proliferative cells at PDL 3 (Fig. S2e), indicating that mTORC1 is activated during preosteoblast senescence.

### mTORC1 activation accelerates senescence in preosteoblasts

**Fig. 2:**
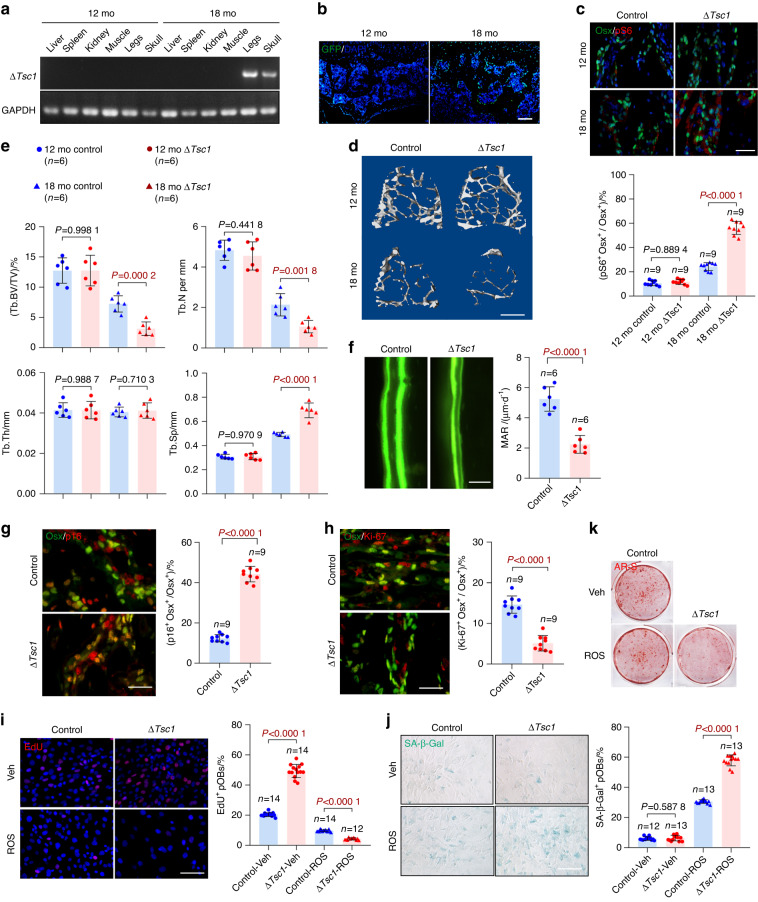
Hyperactive mTORC1 aggravates preosteoblast senescence and age-related bone loss. **a** PCR analysis of *Tsc1* allele recombination in tissues from 12- and 18-month-old Δ*Tsc1* mice. Primers for GAPDH were used as a loading control. **b** Immunostaining of GFP, which indicated Osx-Cre expression, in bone sections of the mice in **a**. Scale bar, 200 μm. **c** Double immunostaining of Osx and pS6 and quantification of pS6^+^ preosteoblasts (pS6^+^ Osx^+^) relative to total preosteoblasts (Osx^+^) in bone sections of the mice in **a**. Scale bar, 100 μm. **d** Representative microcomputed tomography (μCT) images of 12- and 18-month-old Δ*Tsc1* tibias compared with those of the littermate controls, Scale bar, 500 μm. **e** Quantification of Tb. BV/TV, Tb.N, Tb.Sp, and Tb.Th. **f** Representative images of calcein labels and quantification of the mineral apposition rate (MAR) in femurs from the 18-month-old Δ*Tsc1* mice. Scale bar = 50 μm. Double immunostaining of Osx plus p16 (**g**) and Osx plus Ki-67 (**h**) in tibias of the 18-month-old Δ*Tsc1* mice. Double positively stained cells were quantified. Scale bar: 100 μm. In primary calvarial osteoblasts isolated from neonatal Δ*Tsc1* and *Tsc1*^fl/fl^ mice, senescence was induced by ROS. After 3 days, (**i**) the cells were immunostained for EdU and analyzed for EdU^+^ cells relative to total cells. Scale bar, 50 μm. **j** Representative images of SA-β-gal staining and quantification of the proportion of SA-β-gal-positive cells in the senescence-induced cells. Scale bar, 100 μm. **k** After induction of osteogenic differentiation, cells were subjected to AR-S staining on Day 14 after differentiation induction. Data are shown as the mean ± SD. The numbers of samples (*n*) are indicated in each figure panel. *P* values were determined with two-tailed Student’s *t* test for single comparisons

We next investigated the role of mTORC1 activation in preosteoblast senescence by using mice with osteoblast-specific knockout of *Tsc1*, a negative regulator of mTORC1 (Δ*Tsc1* mice). The Δ*Tsc1* mice were fed doxycycline to suppress Osx-Cre expression until they were 12 months old and were sacrificed at the age of 18 months; this treatment resulted in stage-specific preosteoblastic mTORC1 activation at an old age (12–18 months) (Fig. 2a–c). In contrast to the increased bone mass previously observed in young Δ*Tsc1* mice,^16^ more serious bone loss was observed in these Δ*Tsc1* mice at an old age than in the control *Tsc1 flox* mice (Fig. 2d, e). The aged Δ*Tsc1* mice exhibited decreased serum levels of N-terminal propeptide of type 1 collagen (P1NP) (Fig. S3a) and elevated C-terminal telopeptides of type I collagen (CTX-I) (Fig. S4a), indicating impaired bone formation and increased bone resorption in the Δ*Tsc1* mice. Indeed, the numbers of Osx^+^ preosteoblasts (Fig. S3b, c) and mature Ocn^+^ osteoblasts (Fig. S3d, e) were diminished in these old age-specific knockout mice. Double fluorochrome labeling analyses further showed decreased mineral apposition rates (MARs) (Fig. 2f), indicating lower bone formation of osteoblasts in the Δ*Tsc1* mice. The number of osteoclasts, which we have reported to be diminished in young Δ*Tsc1* mice,^16^ was instead elevated in these old Δ*Tsc1* mice (Fig. S4b, c). Immunostaining further revealed elevated p16 expression (Fig. 2g) and proliferative arrest (Fig. 2h) in preosteoblasts of the Δ*Tsc1* mice. These results suggested that preosteoblastic mTORC1 activation aggravates age-related bone loss, possibly by accelerating preosteoblast senescence, thus preventing their differentiation into functional osteoblasts and stimulating osteoclast formation.

To further validate these results in vitro, we isolated primary osteoblasts from calvaria and long bones in the Δ*Tsc1* mice and induced cell senescence with ROS. As expected, ROS-induced increased expression of the senescence marker p16 (Fig. S5a, d), proliferative arrest (Fig. 2i, Fig. S5e), and elevated SA-β-Gal activity (Fig. 2j, Fig. 5f) and expression of SASP components (IL-6 and Cxcl1) (Fig. 5b) in osteoblasts. Moreover, ROS-induced senescence impaired the differentiation potential of osteoblasts (Fig. S5c and Fig. 2k). All these hallmarks of osteoblast senescence were exacerbated by mTORC1 activation in the Δ*Tsc1* osteoblasts (Fig. 2i–k, Fig. S5), thereby suggesting that mTORC1 activation accelerates senescence in preosteoblasts. We further found that mTORC1 activation exerted consistent inhibitory effects on apoptosis in replicative and senescent preosteoblasts (Fig. S6a), which could not explain the aggravated bone loss in the Δ*Tsc1* mice. Together, these results suggested that mTORC1 aggravates age-related bone loss by accelerating preosteoblast senescence.

### mTORC1 inhibition enables preosteoblast escape from senescence

**Fig. 3:**
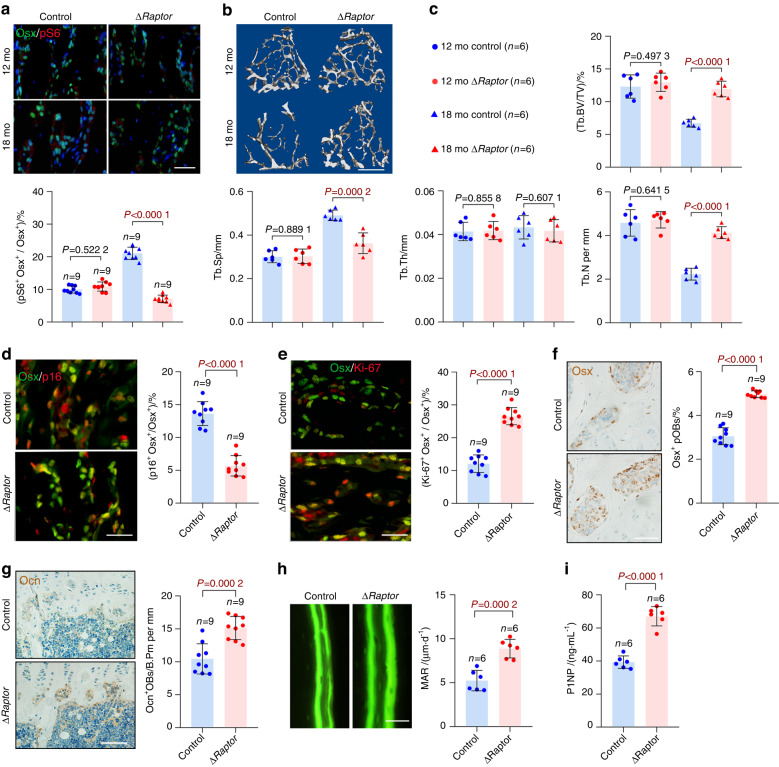
mTORC1 inactivation alleviates preosteoblast senescence and bone loss. **a** Double immunostaining of Osx and pS6 and quantification of pS6^+^ preosteoblasts (pS6^+^ Osx^+^) relative to total preosteoblasts (Osx^+^) in the tibias of 12- and 18-month-old Δ*Raptor* mice. Scale bar, 100 μm. Representative microcomputed tomography (μCT) images (**b**) and quantification of trabecular bone (**c**) in the 12- and 18-month-old Δ*Raptor* tibias compared with those from the littermate controls. Scale bar, 500 μm. Double immunostaining of Osx plus p16 (**d**) and Osx plus Ki-67 (**e**) in tibias. Double positively stained cells were quantified. Scale bars, 100 μm. IHC staining for Osx (**f**) and osteocalcin (Ocn) (**g**) in the tibias of the 18-month-old Δ*Raptor* mice and their littermate controls. Scale bar: 100 μm. Osx-positive preosteoblasts (Osx^+^ pOBs) were quantified as cell numbers per total cells in the bone marrow. Ocn-positive mature osteoblasts (Ocn^+^OBs) on the bone surface were measured as cells per millimeter of perimeter in the sections (/B.Pm). **h** Representative images of calcein labels and quantification of the mineral apposition rate (MAR) in femurs from the 18-month-old Δ*Raptor* mice and their littermate controls. Scale bar = 50 μm. **i** Serum levels of P1NP in the 18-month-old Δ*Raptor* mice and their littermate controls determined by ELISAs. Data are shown as the mean ± SD. The numbers of samples (*n*) are indicated in each figure panel. *P* values were determined by two-tailed Student’s *t* test for single comparisons

To better characterize the roles of mTORC1 in preosteoblast senescence, we established a mouse model with specific mTORC1 inhibition in old age (Fig. 3a) by using the same doxycycline feeding and retreatment procedure as that used in the Δ*Tsc1* mice. Micro-CT analysis revealed increased bone volume in the Δ*Raptor* mice with mTORC1 inactivation in preosteoblasts (Fig. 3b, c). The stimulated bone formation in the Δ*Raptor* mice might have been caused by the alleviated senescence of preosteoblasts (Fig. 3d, e), increased numbers (Fig. 3f, g) and activity (Fig. 3h, i) of osteoblastic lineage cells, and decreased numbers and activity of osteoclasts (Fig. S4).

**Fig. 4:**
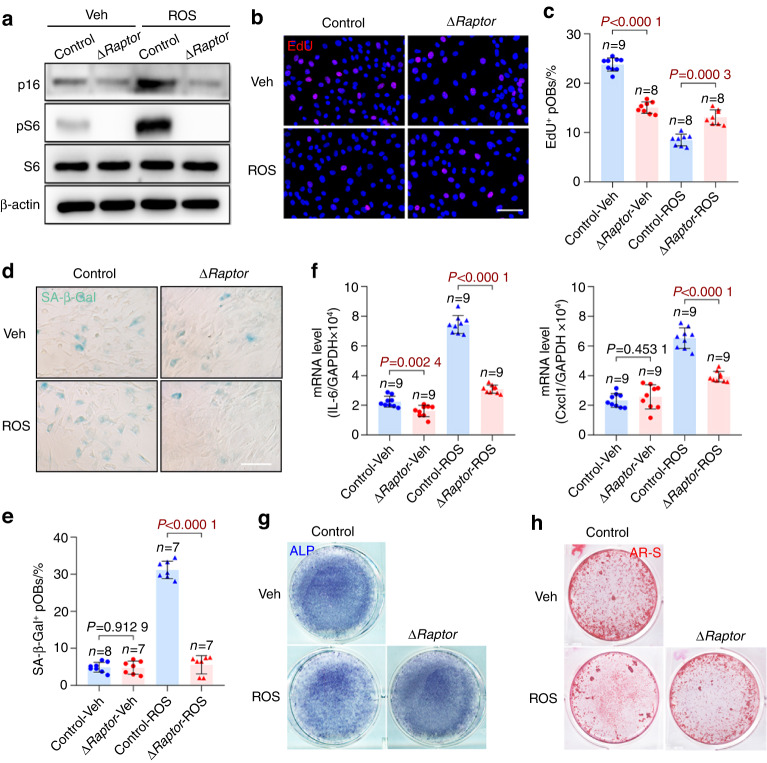
mTORC1 inhibition enables preosteoblast escape from senescence in vitro. **a** Primary calvarial osteoblasts isolated from neonatal Δ*Raptor* and *Raptor*^fl/fl^ mice were induced to undergo senescence by ROS. After 3 days, the cells were analyzed for senescence marker (p16 and p53) expression and mTORC1 activity (pS6) with western blotting. **b** Immunostaining of EdU in the senescence-induced cells in **a**. Scale bar, 50 μm. **c** Quantitative analysis of EdU^+^ cells relative to total cells. **d** Representative images of SA-β-gal staining of the senescence-induced cells in **a**. Scale bar, 100 μm. **e** Quantification of the proportion of SA-β-gal-positive cells in each population. **f** qPCR analysis of IL-6 and Cxcl1 mRNA in the primary preosteoblasts in **a**. The cells in a were then induced to undergo osteogenic differentiation and subjected to (**g**) ALP staining or (**h**) AR-S staining on Day 7 or 14, respectively, after induction of differentiation. Data are shown as the mean ± SD. The numbers of samples (*n*) are indicated in each figure panel. *P* values were determined with two-tailed Student’s *t* test for single comparisons

Consistent with the in vivo results, the Δ*Raptor* preosteoblasts exhibited a slower increase in p16 expression (Fig. 4a, Fig. S5d), less pronounced impairment of the proliferative capacity (Fig. 4b, c, Fig. S5e), lower SA-β-Gal activity (Fig. 4d, e, Fig. S5f), less SASP component secretion (Fig. 4f) and greater differentiation (Fig. 4g) and mineralization (Fig. 4h) when subjected to ROS-induced senescence. Moreover, the Δ*Raptor* preosteoblasts showed consistently elevated apoptosis rates in both the replicative and senescent states (Fig. S6b); this finding could not explain the alleviated bone loss in the aged Δ*Raptor* mice. On the basis of these results, we concluded that mTORC1 inhibition alleviated age-related bone loss by enabling preosteoblast escape from senescence.

### Plasma membrane depolarization induces preosteoblast senescence

**Fig. 5:**
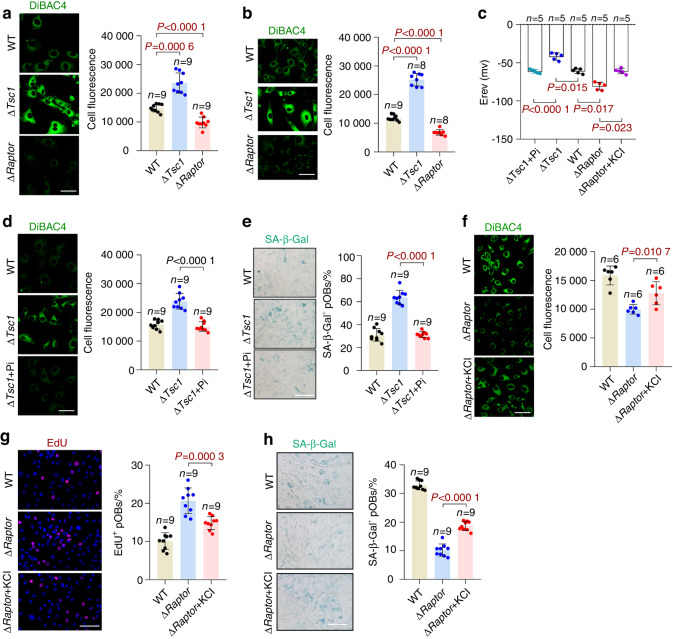
Plasma membrane depolarization contributes to preosteoblast senescence. Senescent wild-type (WT), Δ*Tsc1*, and Δ*Raptor* preosteoblasts isolated from calvaria (**a**) or long bones (**b**) were incubated with fluorescent DiBAC4 dye and photographed under a confocal microscope. Scale bars, 50 μm. Cell fluorescence was measured as the mean corrected total cell fluorescence (CTCF) of all the cells in nine different images taken at ×400 magnification in ImageJ. Depolarization is indicated by increased fluorescence. **c** Histograms showing the mean reversal potentials (E_rev_) of ramp membrane currents determined by the patch-clamp technique in the different indicated preosteoblasts. **d** Representative confocal images of DiBAC4 fluorescence in the senescent Δ*Tsc1* preosteoblasts treated with pinacidil (Pi). Scale bar, 50 μm. The fluorescence intensity of the cells was measured. **e** SA-β-gal staining of the cells in **d** and quantification of the proportion of SA-β-gal-positive cells in each population. Scale bar, 100 μm. **f** DiBAC4 fluorescence in the senescent Δ*Raptor* preosteoblasts treated with KCl. Relative plasma membrane potentials were measured. **g** Immunostaining of EdU in the cells in **f** and quantitative analysis of EdU^+^ cells relative to total cells. Scale bar, 100 μm. **h** SA-β-gal staining of the cells in **f** and quantification of the proportion of SA-β-gal-positive cells. Scale bar, 100 μm. Data are shown as the mean ± SD. The numbers of samples (*n*) are indicated in each figure panel. *P* values were determined by two-tailed Student’s *t* test for single comparisons

We then explored the mechanisms responsible for the regulation of preosteoblast senescence by mTORC1. Recently, membrane depolarization has been reported to be associated with mTORC1 and cell senescence.^19,20^ Therefore, we investigated whether potential changes in the plasma membrane might have a role in mTORC1-regulated preosteoblast senescence. To measure their relative plasma membrane potential, we incubated senescent preosteoblasts with the fluorescent dye DiBAC4, which is increasingly taken up by cells with plasma membrane depolarization (and decreases in hyperpolarized cells). The Δ*Tsc1* preosteoblasts, which presented accelerated senescence, as described above, showed an elevated DiBAC4 fluorescence intensity, whereas the Δ*Raptor* preosteoblasts displayed decreased plasma membrane depolarization (Fig. 5a, b). The patch-clamp experiments revealed the same potential changes in the cells (Fig. 5c).

Interestingly, because depolarization was prevented by pinacidil (Fig. 5c, d), the Δ*Tsc1* preosteoblasts exhibited decreased senescence, as indicated by an increased proliferation rate (Fig. S7a), decreased SA-b-Gal activity (Fig. 5e) and mRNA levels of SASP components (Fig. S7b). We further treated the senescent Δ*Raptor* preosteoblasts with KCl, a well-established plasma membrane depolarizer. Forced depolarization in the Δ*Raptor* preosteoblasts (Fig. 5c, f) abrogated the beneficial effect of mTORC1 inactivation on cell senescence (Fig. 5g, h and Fig. S8). These results suggested that mTORC1 regulates preosteoblast senescence by controlling plasma membrane potential.

### The sodium channel Scn1a mediates plasma membrane depolarization in senescent preosteoblasts

**Fig. 6:**
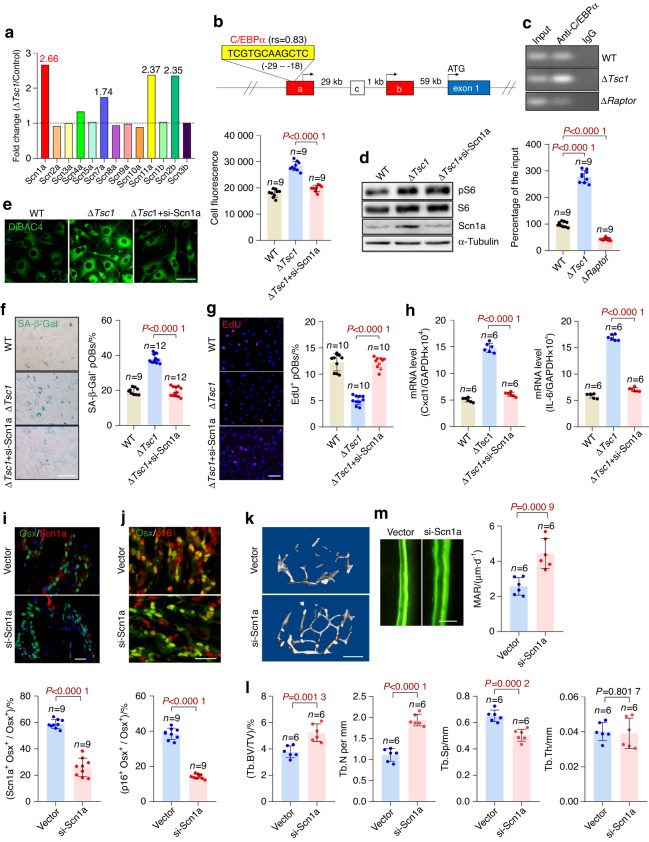
The sodium channel Scn1a mediates plasma membrane depolarization in senescent preosteoblasts. **a** mRNA expression of voltage-sensitive sodium channels in the Δ*Tsc1* and control calvarial preosteoblasts indicated by our previous global mRNA expression profile (GSE74781). **b** Binding site of C/EBPα in the 5′-UE of *Scn1a* (yellow box) predicted by the JASPAR database (http://jaspar.genereg.net). rs: JASPAR relative scores, which are defined as 1 for the maximum-likelihood sequence. Boxes represent exons: blue coding exons, red noncoding exons conserved between humans and mice, white noncoding exons identified in either human or mouse transcripts. Noncoding exons are named alphabetically, and the first coding exon (exon 1) of the *Scn1a* gene is indicated. Genomic distances between exons are indicated. **c** Binding of C/EBPα to the 5′-UE sequence of Scn1a in vivo, determined by a ChIP assay using the Δ*Tsc1* and Δ*Raptor* cells and anti-C/EBPα antibody or IgG. The ChIP samples were then subjected to qPCR with the *Scn1a* 5′-UE primers. The percentage of the input of the sample by using the anti-C/EBPα antibody in the wild-type cells was normalized to 100. The Δ*Tsc1* osteoblasts were transfected with Scn1a siRNA and subjected to (**d**) Scn1a detection with western blotting, (**e**) measurement of relative plasma membrane potential with DiBAC4 dye, (**f**) SA-β-gal staining and quantification of the proportion of SA-β-gal-positive cells, (**g**) immunostaining of EdU and quantitative analysis of EdU^+^ cells relative to total cells, and (**h**) qPCR analysis of IL-6 and Cxcl1 mRNA. Double immunostaining of Osx plus Scn1a (**i**) and Osx plus p16 (**j**) in the tibias of the 18-month-old Δ*Tsc1* mice injected with adenovirus encoding si-Scn1a for 1 month. Double positively stained cells were quantified. Representative μCT images (**k**) and quantification of trabecular bone (**l**) in the mice. (**m**) Representative images of calcein labels and quantification of the mineral apposition rate (MAR) in femurs from the 18-month-old Δ*Tsc1* mice receiving si-Scn1a. Scale bars: 50 μm in **e**, **g**, **m**; 100 μm in **i**, **j**; and 500 μm in **k**. Data are shown as the mean ± SD. The numbers of samples (*n*) are indicated in each figure panel. *P* values were determined with two-tailed Student’s *t* test for single comparisons

We next investigated the mechanisms underlying the regulation of plasma membrane potential by mTORC1. Ion flow through ion channels causes changes in membrane potential. We were particularly interested in voltage-sensitive sodium (Na_v_) channels because they mediate fast membrane depolarization^23^ and have been reported to be associated with cell senescence.^20^ To screen for the Na_v_(s) regulated by mTORC1, we analyzed our previous global mRNA expression profiles of the Δ*Tsc1* and control calvarial osteoblasts (GSE74781).^17^ Scn1a (sodium channel protein type 1 subunit alpha) showed the most significantly increased expression in the Δ*Tsc1* osteoblasts among the Na_v_(s) (Fig. 6a); this result was then verified by quantitative PCR (qPCR) analysis (Fig. S9a). In contrast, Scn1a mRNA levels were lower in the Δ*Raptor* osteoblasts (Fig. S9a). The protein levels of Scn1a were also elevated in the Δ*Tsc1* but diminished in the Δ*Raptor* osteoblasts (Fig. S9b, c), thus indicating positive regulation of Scn1a by mTORC1.

How does mTORC1 regulate Scn1a expression? The transcription factor C/EBPα is known to be positively regulated by mTORC1^24^ and has been shown to bind 5′-untranslated exons (UE) of *SCN1A* and substantially enhance its transcription.^25^ We therefore investigated whether C/EBPα mediates the regulation of Scn1a by mTORC1. Indeed, we found that both the mRNA (Fig. S9d) and protein (Fig. S9e) levels of C/EBPα were elevated in the Δ*Tsc1* but diminished in the Δ*Raptor* osteoblasts. By using transcription factor databases, we predicted the presence of a putative C/EBPα binding site on the 5′-UE of *Scn1a* (Fig. 6b). In ChIP assays, C/EBPα was further demonstrated to bind the 5′-UE of *Scn1a* in mouse preosteoblasts (Fig. 6c). mTORC1 activation promoted and mTORC1 inhibition attenuated this binding (Fig. 6c). Importantly, the increased expression of Scn1a was reversed by siRNA downregulation of C/EBPα expression in the Δ*Tsc1* preosteoblasts (Fig. S9f), thus confirming that C/EBPα is involved in the regulation of Scn1a gene transcription by mTORC1.

We then determined whether Scn1a mediates the regulation of membrane potential and cell senescence by mTORC1. Knockdown of Scn1a expression by siRNA (Fig. 6d) alleviated membrane depolarization (Fig. 6e) and reversed the senescent phenotypes (Fig. 6f–h) in the Δ*Tsc1* preosteoblasts. To validate these results in vivo, we injected the aged Δ*Tsc1* mice with adenovirus encoding si-Scn1a. With interference with Scn1a expression in preosteoblasts (Fig. 6i), the Δ*Tsc1* mice exhibited alleviated preosteoblast senescence (Fig. 6j) and increased bone mass (Fig. 6k, l). Histomorphometric measurements further showed increased mineral apposition rates (MARs) indicative of higher bone formation activity of osteoblasts in the Δ*Tsc1* mice receiving si-Scn1a (Fig. 6m). These results demonstrated that Scn1a functionally mediated plasma membrane potential changes and cellular senescence in preosteoblasts.

### Prosenescence stress activates Scn1a by inhibiting its phosphorylation by PKA

**Fig. 7:**
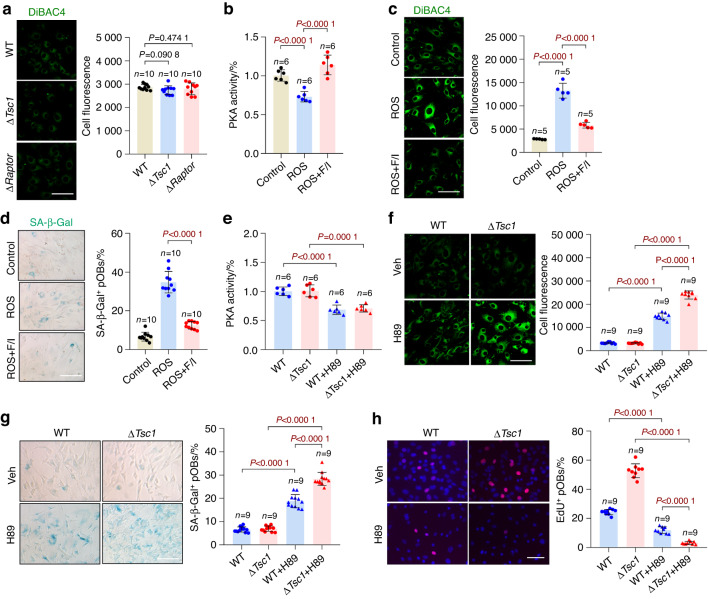
Prosenescent stress activates Scn1a by inhibiting PKA. **a** Replicative wild-type (WT), Δ*Tsc1* and Δ*Raptor* calvarial osteoblasts were incubated with the fluorescent DiBAC4 dye and photographed under a confocal microscope. Scale bar, 50 μm. Relative plasma membrane potentials were measured. ROS-induced senescent wild-type preosteoblasts were treated with F/I (forskolin + IBMx, PKA activator) or left untreated. The cells were subjected to (**b**) measurement of total cellular PKA activities, (**c**) DiBAC4 staining and measurement of relative plasma membrane potentials. Scale bar, 50 μm. **d** SA-β-gal staining of cells in b and quantification of the proportion of SA-β-gal-positive cells in each population. Scale bar, 100 μm. Replicative WT and Δ*Tsc1* osteoblasts were treated with H-89 (PKA inhibitor) and were subjected to (**e**) measurement of total cellular PKA activities, (**f**) DiBAC4 staining and measurement of relative plasma membrane potentials. Scale bar, 50 μm. **g** SA-β-gal staining of cells in **e** and quantification of the proportion of SA-β-gal-positive cells in each population. Scale bar, 100 μm. **h** Immunostaining of EdU in cells in **e** and quantitative analysis of EdU^+^ cells relative to total cells. Scale bar, 100 μm. Data are shown as the mean ± SD. The numbers of samples (*n*) are indicated in each figure panel. *P* values were determined by two-tailed Student’s *t* test for single comparisons

Interestingly, we further found that the regulation of membrane depolarization by mTORC1 was confined to senescent osteoblasts, because mTORC1 activation or inhibition did not cause a significant change in membrane potential in replicative osteoblasts without senescence induction (Fig. 7a). This result suggested that the permeability of Scn1a to Na^+^ might be controlled by prosenescence stress. The function of Scn1a in mediating sodium currents is suppressed by its phosphorylation by the protein kinase PKA.^26–28^ Coincidentally, PKA activity has been reported to be diminished in senescent cells.^29^ Therefore, we reasoned that prosenescent stress might activate Scn1a by inhibiting its phosphorylation through suppression of PKA. As expected, PKA activity was found to decrease in senescent preosteoblasts (Fig. 7b). PKA activation by F/I (forskolin + IBMx) (Fig. 7b) abolished the membrane depolarization (Fig. 7c) and senescent phenotype (Fig. 7d and Fig. S10) of preosteoblasts induced by ROS. In contrast, the PKA inhibitor H-89 alone was sufficient to induce membrane depolarization (Fig. 7e, f) and senescence (Fig. 7g, h and Fig. S11) in preosteoblasts. Moreover, the Δ*Tsc1* preosteoblasts exhibited greater depolarization (Fig. 7f) and more significant senescent phenotypes (Fig. 7g, h and Fig. S11) than the wild-type preosteoblasts after treatment with H-89. These results verified that prosenescent stress activates Scn1a by inhibiting its phosphorylation through suppression of PKA, thereby explaining the unique state of membrane depolarization in senescence and the age-specific effects of mTORC1 on osteoblasts in old mice.

### Plasma membrane depolarization increases Ca^2+^ influx and activates NFAT/ATF3/p53 signaling, thereby inducing preosteoblast senescence

**Fig. 8:**
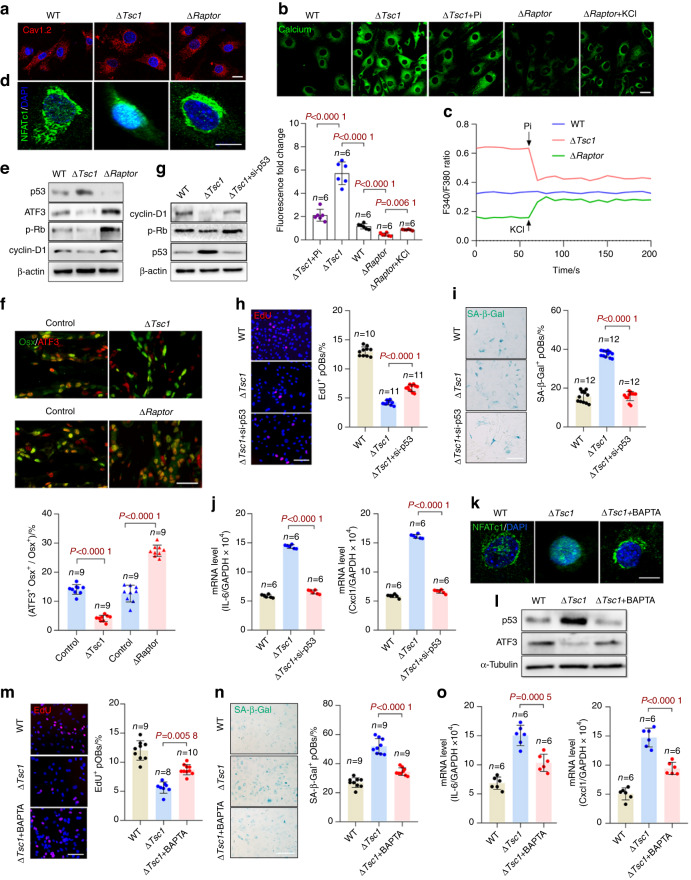
Plasma membrane depolarization induces preosteoblast senescence via the Ca^2+^/NFAT/ATF3/p53 signaling pathway. **a** Representative images of immunostaining of the voltage-gated calcium channel Ca_v_1.2 in primary preosteoblasts. Scale bar, 20 μm. **b** Senescent Δ*Tsc1* and Δ*Raptor* preosteoblasts were treated with pinacidil (Pi) or KCl and incubated with the FluoForte probe to measure the relative cytosolic calcium levels. Calcium imaging data were quantified by normalization of the values to those of senescent wild-type (WT) preosteoblasts. Scale bar, 20 μm. **c** Representative Ca^2+^ traces for the senescent Δ*Tsc1* and Δ*Raptor* preosteoblasts with added pinacidil or KCl. **d** Immunostaining of NFATc1 and (**e**) western blot analysis of ATF3, p53, cyclin D1, and Rb phosphorylation levels in the senescent Δ*Tsc1* and Δ*Raptor* preosteoblasts. Scale bar, 10 μm in **d**. **f** Immunostaining of ATF3 and Osx in the Δ*Tsc1* and Δ*Raptor* mouse bone and quantitative analysis of the ATF3^+^ preosteoblasts (ATF3^+^ Osx^+^) relative to the total Osx^+^ preosteoblasts. Scale bar, 100 μm. **g** Western blot analysis of cyclin D1 expression and Rb phosphorylation in the Δ*Tsc1* preosteoblasts with interference with p53 expression. **h** Immunostaining of EdU in cells in g and quantitative analysis of EdU^+^ cells relative to total cells. Scale bar, 100 μm. **i** SA-β-gal staining of the cells in g and quantification of the proportion of SA-β-gal-positive cells in each population. Scale bar, 100 μm. **j** qPCR analysis of Cxcl1 and IL-6 mRNA in the cells in **g**. **k** Immunostaining of NFATc1 in the senescent Δ*Tsc1* preosteoblasts treated with BAPTA-AM (a calcium chelator) or left untreated. Scale bar, 10 μm. **l** Western blot analysis of ATF3 and p53 expression in the cells in **k**. **m** Immunostaining of EdU in the cells in **k** and quantitative analysis of EdU^+^ cells relative to total cells. Scale bar, 100 μm. **n** SA-β-gal staining of the cells in **k** and quantification of the proportion of SA-β-gal-positive cells in each population. Scale bar, 100 μm. **o** qPCR analysis of Cxcl1 and IL-6 mRNA in the cells in **k**. Data are shown as the mean ± SD. The numbers of samples (*n*) are indicated in each figure panel. *P* values were determined by two-tailed Student’s *t* test for single comparisons

Finally, we investigated the mechanisms through which plasma membrane depolarization contributes to preosteoblast senescence. Plasma membrane depolarization is known to activate voltage-gated calcium channels and increase intracellular calcium,^30^ and this increased calcium can promote cell senescence.^31,32^ Indeed, Ca_v_1.2 was expressed constitutively in preosteoblasts (Fig. 8a). Moreover, the senescent Δ*Tsc1* preosteoblasts displayed elevated cytosolic calcium levels, which were then decreased by the repolarization induced by pinacidil. In contrast, calcium levels were diminished in the senescent Δ*Raptor* preosteoblasts but were increased by KCl-induced depolarization (Fig. 8b, c). Because Ca_v_1.2 showed consistent expression among the two sets of transgenic preosteoblasts and the wild-type controls (Fig. 8a), we speculated that plasma membrane depolarization might increase Ca^2+^ influx by activating calcium channels.

Increased cytosolic calcium induces cell senescence by triggering NFAT dephosphorylation and its translocation to the nucleus.^33^ NFATc1 inhibits the expression of ATF3, which in turn suppresses the expression of p53 and other senescence-related markers.^34^ p53 induces transcription of the cyclin-dependent kinase inhibitor p21. In turn, p21 blocks CDK4/6 activity, thus resulting in decreased cyclin D levels, hypophosphorylated Rb and cell cycle exit.^35^ Consistent with these findings, we observed that translocation of NFATc1 to the nucleus was promoted in the senescent Δ*Tsc1* preosteoblasts (Fig. 8d). Consequently, ATF3 expression was diminished in the Δ*Tsc1* preosteoblasts in vitro (Fig. 8e) and in bone (Fig. 8f), thereby further increasing p53 expression and decreasing cyclin D levels and Rb phosphorylation in the cells (Fig. 8e). In contrast, the senescent Δ*Raptor* cells exhibited inhibited NFAT/ATF3/p53 signaling and increased cyclin D levels and Rb phosphorylation (Fig. 8d–f). After interference with p53 expression, the Δ*Tsc1* preosteoblasts showed increased cyclin D1 levels and Rb phosphorylation (Fig. 8g) and alleviated senescent phenotypes (Fig. 8h–j). Moreover, the addition of a calcium chelator (BAPTA-AM) abolished the activation of NFATc1/ATF3/p53 signaling (Fig. 8k, l) and the increase in senescence markers (Fig. 8m–o) in the Δ*Tsc1* preosteoblasts. These results suggested that plasma membrane depolarization causes preosteoblast senescence by increasing Ca^2+^ influx and activating NFAT/ATF3/p53 signaling.

## Discussion

**Fig. 9:**
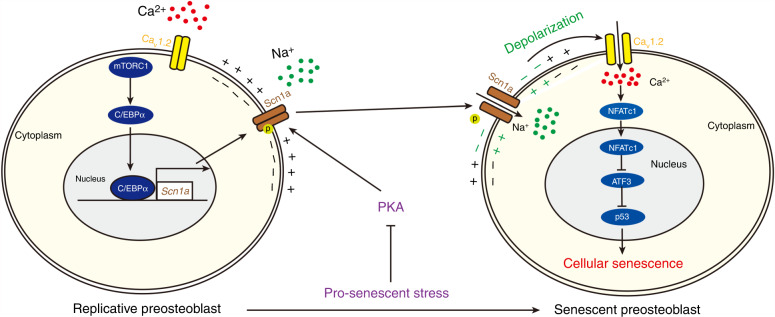
Model of the sodium channel Scn1a in regulating preosteoblast senescence. Scn1a expression is positively regulated by mTORC1 upstream of C/EBPα, whereas its permeability to Na^+^ is gated by protein kinase A (PKA)-induced phosphorylation. Prosenescent stresses increase the permeability of Scn1a to Na^+^ by suppressing PKA activity and induce depolarization in preosteoblasts. Depolarization increases Ca^2+^ influx and activates downstream NFAT/ATF3/p53 signaling, thus promoting preosteoblast senescence

Understanding the mechanisms responsible for preosteoblast senescence is critical for the development of therapeutic interventions for age-related bone loss. Using mouse models with mTORC1 activation or inhibition in preosteoblasts during old age, we identified the sodium channel Scn1a as a novel regulator of preosteoblast senescence. We found that the plasma membranes in senescent preosteoblasts are depolarized, which promotes cell senescence by increasing Ca^2+^ influx and activating downstream NFAT/ATF3/p53 signaling. Scn1a promotes the senescence of preosteoblasts by driving depolarization of the plasma membrane. Scn1a expression is positively regulated by mTORC1 upstream of C/EBPα, whereas its permeability to Na^+^ is tightly regulated by PKA activity during cell senescence. Prosenescent stresses elevate the permeability of Scn1a to Na^+^ by suppressing PKA activity and induce depolarization specifically in senescent preosteoblasts (Fig. 9). Thus, Scn1a is a novel regulator of preosteoblast senescence during age-related bone loss.

For more than 100 years, cells have been known to have a transmembrane potential,^18^ which is maintained by the balance between ions on both sides of the plasma membrane. Whereas early studies on membrane potential primarily focused on excitability, scientists have recently shown that dynamic membrane potential also exists in most nonexcitable cells, although its role is unclear. Emerging studies indicate that membrane potential is involved in a wide range of biological functions of cells, including the cell cycle of T cells,^36^ proliferation of vascular smooth muscle cells,^37^ volume control of chondrocytes,^38^ secretion of pancreatic β cells,^39^ migration of epithelial cells^40^, and circadian rhythm of fibroblasts.^41^ Lallet-Daher et al. first reported alterations of plasma membrane potential in cell senescence.^42^ Thereafter, the senescence of fibroblasts^19^ and epithelial cells^20^ was ascribed to membrane potential changes in several studies, although the responsible mechanisms are unclear. Here, we showed that depolarization induces preosteoblast senescence by increasing Ca^2+^ influx and activating downstream NFAT/ATF3/p53 signaling.

Voltage-gated calcium (Ca^2+^) channels (VGCCs) are key transducers of membrane potential changes into intracellular Ca^2+^ transients.^43^ We found that Ca_v_1.2 is constitutively expressed by preosteoblasts and might mediate the Ca^2+^ influx caused by depolarization. Moreover, Fei et al. found that Ca_v_1.2 per se facilitates the osteogenesis of bone marrow-derived mesenchymal stem cells, and upregulation of Ca_v_1.2 expression alleviated osteoporosis in premature aging mice.^44^ However, Ca_v_1.2 exhibited consistent expression among the two sets of transgenic preosteoblasts and the wild-type controls in the present study. Therefore, the increased Ca^2+^ influx in the *ΔTsc1* preosteoblasts was due to elevated Ca^2+^ channel activation after membrane depolarization in the cells.

Beyond increasing Ca^2+^ influx, depolarization might exert other adverse effects on senescent cells. For example, depolarization elevates intracellular Na^+^, which compromises Na^+^-dependent nutrient transport into cells. Coincidentally, senescent cells have been reported to be defective in nutrient sensing.^19^ Given the various adverse effects on cells and the possibility of reversible and rapid modulation, the membrane potential of osteoblasts is a promising target for the development of safe and rapid response drugs for the treatment of age-related bone loss.

The present study revealed that the sodium channel Scn1a mediates membrane depolarization in senescent preosteoblasts. The Scn1a gene encodes the α subunit of the voltage-gated sodium transmembrane protein Na_v_1.1, which allows sodium ions to flow into cells, causes membrane depolarization and induces action potentials in excitable cells.^45^ Na_v_1.1 consists of an α subunit and two β subunits.^46^ In the present study, the expression of the α subunit (Scn1a), but not the β subunits, was revealed to be regulated by mTORC1 during preosteoblast senescence. Moreover, knockdown of Scn1a was sufficient to alleviate membrane depolarization in the senescent Δ*Tsc1* preosteoblasts. On the basis of these observations, we inferred that the α subunit alone was sufficient to induce sodium channel function in preosteoblasts. In support of this possibility, the Na-channel α subunit has been found to act as a channel in oocytes independently of the β subunits.^47–49^

Despite the important roles of Scn1a in membrane depolarization and related biological processes, little is currently known about the machinery underlying the regulation of Scn1a transcription. Voltage-gated sodium channels generally have complex 5′ UTRs consisting of multiple alternatively spliced noncoding exons distributed over large genomic intervals.^23^ Among the sodium channels, SCN1A has the most complex 5′ UTR organization, with seven noncoding exons distributed over a 75-kb interval upstream of exon 1.^50^ These 5′-UEs, with 70 putative transcription factor-binding sites, greatly enhance Scn1a gene expression.^25^ In the present study, we found that Scn1a transcription was positively regulated by mTORC1. Because C/EBPα is known to act downstream of mTORC1^24,51^ and has been predicted to bind the 5′-UE of Scn1a,^25^ we further investigated whether C/EBPα might mediate the positive regulation of Scn1a by mTORC1. Indeed, we observed binding of C/EBPα to the 5′-UE of Scn1a in osteoblasts, and this binding was promoted by mTORC1 activation but attenuated by mTORC1 inhibition. In addition, downregulation of C/EBPα expression reversed the increased expression of Scn1a by mTORC1 activation, thus clearly demonstrating that C/EBPα mediates the positive regulation of Scn1a expression by mTORC1.

mTORC1 is well known for its beneficial roles in promoting development and growth.^52,53^ Later in life, however, mTORC1 exerts detrimental effects by driving senescence and increasing the risk of disease.^15^ The age-specific roles of mTORC1 conform well to the antagonistic pleiotropy hypothesis, which suggests that aging is a byproduct of an investment in development and reproduction and that genetic variants favored in the fertile stages can cause senescence later in life.^54,55^ mTORC1 also appears to exert age-specific effects on bone. We previously revealed a positive effect of mTORC1 on preosteoblast expansion and bone formation during bone development,^16,17^ whereas the present study further uncovered the adverse effects of mTORC1 on preosteoblasts and bone mass maintenance in old age.

mTORC1-driven cell hyperproliferation and anabolism are generally presumed to cause aging.^56^ However, we found that mTORC1 activation-induced preosteoblast senescence does not require mTORC1-driven overgrowth in earlier stages because mTORC1 was not activated at the early ages in the mice in the present study. Instead, the distinct roles of mTORC1 on osteoblast and bone formation in old age are ascribed to the unique membrane depolarization in senescent preosteoblasts. mTORC1 controls membrane potential by regulating the expression of the sodium channel Scn1a; however, the opening probability of Scn1a remains inhibited by PKA-induced phosphorylation.^26,57^ The increased Scn1a by mTORC1 activation does not evoke depolarization in the replicative Δ*Tsc1* preosteoblasts during bone development because most Scn1a channels are likely to be closed owing to phosphorylation by excess PKA activity. In the preosteoblasts of aged mice, however, most Scn1a channels are open, thus causing depolarization and inducing cell senescence in the presence of the decreased PKA activity resulting from prosenescent stress.^29^ Consistently, Lallet-Daher et al. also demonstrated decreased PKA activity in senescent epithelial cells, which induces cell senescence by activating the potassium channel KCNA1 and altering the membrane potential.^42^ PKA appears to exert central roles in the membrane potential changes during cell senescence by gating control of ion channels, thus providing an explanation for the age-specific effects of mTORC1 on preosteoblasts and shedding new light on the antagonistic pleiotropy of mTORC1.

In conclusion, we reveal the mechanisms underlying mTORC1/Scn1a/plasma membrane depolarization-induced osteoblast senescence, including the transcriptional regulation of Scn1a by mTORC1, gating control of Scn1a by prosenescent stress, and the involvement of Scn1a, plasma membrane depolarization and calcium in the regulation of the NFAT/ATF3/p53 pathway. Hence, this work provides new insights into the involvement of ion channels and plasma membrane potential in the control of preosteoblast senescence. Pharmaceutical studies of the related pathways and agents might lead to novel potential treatments for age-related bone loss.

## Materials and methods

### Mice

Transgenic Osx-cre [006361-B6.Cg-Tg(Sp7-tTA,tetO-EGFP/cre)1Amc/J], *Tsc1*^flox/flox^ (005680-STOCK *Tsc1*^tm1Djk^/J) and *Raptor*^flox/flox^ (013188-B6.Cg**-***Rptor*^*tm1.1Dmsa*^/J) mouse strains were purchased from the Jackson Laboratory. The background of the *Tsc1*^flox/flox^ mice was 129s4/SvJae, and these mice were backcrossed to a C57BL/6 background for eight generations before use. Genotyping of these mice was conducted with genomic DNA isolated from tail biopsies, with the primers listed in Table S1. To prevent the Osx promoter from driving Cre expression, we exposed the mice to 200 μg·mL^−1^ doxycycline (Sigma-Aldrich) in their drinking water. C57BL/6J (wild-type) mice were purchased from the Experimental Animal Center of Southern Medical University (Guangzhou, China).

### Micro-CT analysis

The three-dimensional structure of trabecular bone was analyzed in the femora or tibias on a micro-CT scanner (Viva CT40; Scanco Medical AG, Bassersdorf, Switzerland). For the femora, scanning was started from the lower growth plate and extended proximally for 300 slices at 12-μmol·L^−1^ resolution. Morphometric analysis was conducted with the first slice in which the femoral condyles were fully merged and extended for 100 slices proximally. For tibias, scanning was performed in the proximal metaphysis, starting from an anatomic landmark in the growth plate and extending 300 slices distally.

Morphometric analysis was conducted with the first slice in which the growth plate disappeared and was extended for 100 slices distally. The trabecular bones of the femora and tibias were segmented from the cortical shell manually on key slices by using a contouring tool, and then, the contours were morphed automatically to segment the trabecular bone on all slices. The three-dimensional structure and morphometry were constructed and analyzed for trabecular bone volume fraction (BV/TV), trabecular thickness (Tb.Th), trabecular number (Tb.N) and trabecular separation (Tb.Sp). Two-dimensional images were used to generate 3D reconstructions in the image processing software Materialize Mimics (Materialize NV, Belgian).

### Immunohistochemistry and histochemistry staining

Mouse tibial tissues were fixed with 4% paraformaldehyde in PBS at 4 °C for 48 h and then 10% EDTA (pH 8.0) at 4 °C for 14 days. The tissues were embedded in paraffin or optimal cutting temperature compound (Sakura Finetek), and 3–5 μm sagittally oriented sections were prepared for histological analyses. For immunohistochemistry, we incubated primary antibodies that recognized mouse phospho-S6 (Ser235/236) (Cell Signaling, 1:100, #2211), Osterix (Abcam, 1:200, ab22552), osteocalcin (Abcam, #ab93876), p16 (Santa Cruz Biotechnology, 1:200, #sc-1661) and Scn1a (Boster Biological Technology, 1:200, #PB0932) overnight at 4 °C. TRAP staining (Sigma-Aldrich, Missouri, USA) was performed according to the manufacturer’s instructions. Cells per bone perimeter (B.Pm) or the percentage of positive cells was used to calculate the number of positive cells. At least three mice per group were examined. Three equidistant sections spaced 200 µm apart throughout the midsagittal sections of the tibia were evaluated.

### Cell culture and treatment

We purchased the mouse preosteoblast cell line MC3T3-E1 from the American Type Culture Collection. The cells were maintained in α-minimal essential Eagle’s medium (αMEM, Corning) containing 10% FBS (Gibco) at 37 °C with 5% CO_2_. Calvarial osteoblastic cells were prepared from the calvariae of newborn mice (24 h after birth), washed with PBS and digested in 0.1 mg·mL^−1^ collagenase type II (Thermo Fisher Scientific, #17101015) in αMEM at 37 °C for 20 min with five replicates. The supernatants were then combined and centrifuged to pellet the cells after digestion. We also isolated osteoblastic cells from the long bones of mice as described previously.^58^ Primary osteoblastic cells were cultured in αMEM supplemented with 10% FBS and 100 U·mL^−1^ penicillin/streptomycin (Invitrogen) and then incubated with tert-butyl hydroperoxide (t-BHP) (Sigma-Aldrich, 100 µmol·L^−1^), pinacidil (MedChemExpress, 100 µmol·L^−1^, #HY-14290A), H-89 (Beyotime Institute of Biotechnology, 10 µmol·L^−1^, #S1643), forskolin (Meilunbio, 10 µmol·L^−1^, #MB5959), IBMX (Meilunbio, 100 µmol·L^−1^, #MB5226) or BAPTA-AM (MedChemExpress, 20 µmol·L^−1^, #HY-100545) treatment as indicated.

For ROS-induced cell senescence, preosteoblasts were incubated with tert-butyl hydroperoxide (t-BHP) (Sigma-Aldrich, 100 µmol·L^−1^, #458139) for three consecutive days. To induce osteogenic differentiation, we plated primary osteoblastic cells at a density of 1.5 × 10^5^ cells/well (six-well plates) and incubated them with osteoblast differentiation medium with β-glycerophosphate (Sigma-Aldrich, 5 mmol·L^−1^) and ascorbic acid (Sigma-Aldrich, 50 µg·mL^−1^).

### Cell staining

For SA-β-galactosidase staining, cells were washed with PBS, fixed with 4% paraformaldehyde in PBS at room temperature for 20 min and incubated with reagents from a senescence-associated β-galactosidase staining kit (Beyotime Institute of Biotechnology, #C0602) according to the manufacturer’s suggestions.

For alkaline phosphatase staining, differentiated osteoblasts were washed with PBS, fixed with 4% paraformaldehyde for 30 min at room temperature and stained with an Alkaline Phosphatase Staining Kit (Beyotime Institute of Biotechnology) for 1 h at room temperature in the dark.

For alizarin red staining, cells were fixed with paraformaldehyde for 30 min, incubated with 1% alizarin red for 30 min at room temperature and washed with PBS to remove the excess dye.

For immunocytochemical staining, we incubated cultured cells with primary antibodies recognizing mouse Ca_v_1.2 (Abbkine, 1:100, #Abp57305) or NFATc1 (Santa Cruz Biotechnology, 1:100, #sc-7294) overnight at 4 °C. Secondary antibodies conjugated with fluorescent tags were incubated at room temperature for 1 h in the dark.

### siRNA infection

The siRNAs targeting mouse *Scn1a* and *C/EBPα* were designed and synthesized by GenePharma Co., Ltd. (Shanghai, China). The sequences of Scn1a and C/EBPα siRNA were as follows: si-Scn1a, 5′-GCCUGUCAUUGAACCAGAATT-3′; si-C/EBPα, 5′-GAGCCGAGAUAAAGCCAAATT-3′. Adenovirus encoding siRNA targeting mouse *Scn1a* was produced by Dongze Biotech Co., Ltd. (Guangzhou, China).

We transiently transfected cells with siRNA by using Lipofectamine 3000 (Invitrogen, Carlsbad, CA, USA) in Opti-MEM (Invitrogen) according to the manufacturer’s instructions. The efficiency of transfection was measured by western blotting. Adenoviruses (2 × 10^9^ PFU per mouse) were applied to mice once every 3 days for 1 month via tail-vein injection.

### Western blotting

Cell lysis buffer containing 2% SDS, 2 mol·L^−1^ urea, 10% glycerol, 10 mmol·L^−1^ Tris-HCl (pH 6.8), 10 mmol·L^−1^ dithiothreitol and 1 mmol·L^−1^ phenylmethylsulfonyl fluoride was added to lyse adherent cells. The lysates were centrifuged, and the supernatants were separated by SDS–polyacrylamide gel electrophoresis and blotted onto a PVDF membrane (Merck Millipore, ISEQ00010). The membrane was then incubated with specific antibodies against phospho-S6 (S235/236) (Cell Signaling Technology, 1:1 000, #2211), S6 (Santa Cruz Biotechnology, 1:4 000, #sc-74459), p16 (Santa Cruz Biotechnology, 1:2 000, sc-1661), ATF3 (Abbkine, 1:1 000, #Abp55330), p53 (Cell Signaling Technology, 1:2 000, #2524), Scn1a (Boster Biological Technology, 1:1 000, #PB0932) and C/EBPα (Cell Signaling Technology, 1:2 000, #2295). The membrane was then visualized by enhanced chemiluminescence (ECL Kit, Amersham Biosciences).

### Real-time quantitative PCR

Total RNA was isolated from cell pellets with TRIzol Reagent (TaKaRa Biotechnology, #9109). cDNA was reverse transcribed from RNA samples with reverse transcription reagents (TaKaRa Biotechnology, #RR036A), and qPCR assays were performed with Real-Time PCR Mix (Vazyme Biotech, #Q311–02) in a Light Cycler (Roche Molecular Biochemicals, Indianapolis, IN, USA). Primer sequences are listed in Table S1. The relative quantification of gene expression was performed with the comparative threshold (C_T_) method. Changes in mRNA expression levels were calculated after normalization to values for the GAPDH calibrator gene.

### Cell proliferation assays

A kFluor555-EdU cell proliferation detection kit (Keygen Biotechnology, Jiangsu, China; #KGA337) was used to detect cell proliferation according to the manufacturer’s instructions. Briefly, cells were seeded in 96-well plates (6 000 cells/well) and incubated with 50 μmol·L^−1^ EdU at 37 °C and 5% CO_2_ for 3 h. After fixation with 4% paraformaldehyde for 20 min at room temperature, the cells were stained with a kFluor555 reaction mixture for immunocytochemical analysis. Nine areas in each group were counted by two independent observers blinded to the groups.

### Chromatin immunoprecipitation (ChIP) assays

ChIP assays were conducted with a ChIP assay kit (Cell Signaling Technology) according to the manufacturer’s instructions. Approximately 2 × 10^7^ cells were incubated with 1% formaldehyde to crosslink the protein and DNA. After a 10-min incubation, a final concentration of 0.125 mol·L^−1^ glycine was added to the 1% formaldehyde-PBS solution for neutralization. The harvested cells were lysed, and genomic DNA was sonicated to decrease the DNA length to 400 to 800 bp. The sheared chromatin was immunoprecipitated with 4 μg anti-C/EBPα (Proteintech, 18311–1-AP) or normal IgG as a negative control. Isolated DNA was subjected to PCR both before and after chromatin immunoprecipitation with primers designed to amplify the region within the Scn1a 5′-UE containing the C/EBPα binding site. The primers are listed in Table S1. The PCR products were assessed on 1.0% agarose gels and confirmed by direct sequencing. qPCR was performed according to the above description.

### Plasma membrane potential measurement

Plated cells were washed with PBS and incubated with 200 nmol·L^−1^ of the membrane voltage-reporting dye DiBAC4 (Molecular Probes) in PBS at 37 °C for 1 h. Then, the cells were observed and photographed with a FluoView FV1000 confocal microscope (Olympus). The fluorescence data were analyzed with Image-Pro Plus software.

Alternatively, plasma membrane potential was evaluated with the conventional whole-cell configuration of the patch-clamp technique at room temperature. The internal solution (pH = 7.2) contained 130 mmol·L^−1^ KCl, 1 mmol·L^−1^ MgCl_2_, 3 mmol·L^−1^ MgATP and 5 mmol·L^−1^ HEPES. The external solution (pH = 7.4) contained 140 mmol·L^−1^ NaCl, 4 mmol·L^−1^ KCl, 2 mmol·L^−1^ MgCl_2_, 2.5 mmol·L^−1^ CaCl_2_, 10 mmol·L^−1^ glucose and 10 mmol·L^−1^ HEPES. Membrane currents were evoked from a holding potential of −80 mV by voltage ramps of 1.5-s duration, applied from −100 to +90 mV, sampled at 1 kHz, and filtered at 300 Hz. Reversal potentials of ramp membrane currents were determined by linear fits extrapolated on-ramp currents in a potential range around the zero current and were verified in the I0 current-clamp mode of the patch-clamp amplifier to be close to the membrane potentials of cells.

### Cytosolic calcium level measurements and PKA enzymatic activity assays

Cytosolic calcium levels were detected with a FluoForte^®^ Calcium Assay Kit (Enzo Life Sciences, #ENZ-51017) and Fura-2/AM (Invitrogen™, # F1221) according to the manufacturer’s suggested procedure. Briefly, cells plated in 96-well plates (40 000 cells/well) were incubated with 100 μL of FluoForte^®^ Dye-loading solution at 37 °C for 1 h. Then, the cells were photographed with a FluoView FV1000 confocal microscope (Olympus). Fluorescence micrographs were digitalized, and the results are expressed as the change in fluorescence over baseline fluorescence.

Preosteoblasts were further loaded with 5 μmol·L^−1^ Fura-2/AM in Hanks’ balanced salt solution (HBSS) for 1 h at 37 °C. After the cells were washed extensively with HBSS, cytosolic Ca^2+^ was measured with a calcium imaging system built on an inverted fluorescence microscope (Olympus IX51). Fluorescence images (filtered at 515 nm ± 25 nm) were captured with a CCD camera (CoolSNAP fx-M) and analyzed in MetaFluor software. Ca^2+^ levels are shown as the ratio of fluorescence intensity at 340 nm/fluorescence intensity at 380 nm (F340/F380). At least three independent experiments were performed for each condition.

Total cellular and mitochondrial PKA activities were measured with a nonradioactive PKA Kinase Activity Assay Kit (#ab139435, Abcam) according to the manufacturer’s instructions.^59^

### Statistical analysis

All results are presented as the mean ± SD. Curve analysis was performed in Prism (GraphPad). The data in each group were analyzed with unpaired, two-tailed Student’s *t* test. The significance threshold was set at *P* < 0.05.

## Supplementary information

Supplemental Information

## Data Availability

The authors declare that all data supporting the findings of this study are available. within the article and its Supplementary Information files or from the corresponding author upon reasonable request. The global mRNA expression profile reported in this study has been deposited in the Gene Expression Omnibus (GEO) database under the accession code GSE74781.
