# Correction: mTORC1 induces plasma membrane depolarization and promotes preosteoblast senescence through regulating the sodium channel Scn1a

**DOI:** 10.1038/s41413-023-00276-7

**Published:** 2023-07-20

**Authors:** Ajuan Chen, Jian Jin, Shasha Cheng, Zezheng Liu, Cheng Yang, Qingjing Chen, Wenquan Liang, Kai Li, Dawei Kang, Zhicong Ouyang, Chenfeng Yao, Xiaochun Bai, Qingchu Li, Dadi Jin, Bin Huang

**Affiliations:** 1 Academy of Orthopedics, Guangdong Province, Guangdong Provincial Key Laboratory of Bone and Joint Degeneration Diseases, Department of Spine Surgery, The Third Affiliated Hospital of Southern Medical University, Guangzhou, China; 2 Department of Spine Surgery, Nanfang Hospital, Southern Medical University, Guangzhou, China; 3 Department of Clinical Laboratory, The Third Affiliated Hospital of Southern Medical University, Guangzhou, China; 4 Department of Cell Biology, School of Basic Medical Science, Southern Medical University, Guangzhou, China

**Keywords:** Osteoporosis, Bone

Correction to: *Bone Research* 10.1038/s41413-022-00204-1, published online 08 March 2022

During a reread of our previously published original article^1^, the authors regrettably found that the representative micro-CT images of the femurs in Fig. 1a and Fig. 6k and the tibias in Fig. 2d and Fig. 3b were mistakenly pasted during images assembly. We also noticed that the ‘12 mo ΔTsc1’ at the right side in X axis is incorrectly labelled in Fig. 2c. The correct label should read ‘18 mo ΔTsc1’.

Similarly, the ‘12 mo control’ and ‘12 mo ΔTsc1’ in the second row in Fig. 2e should read ‘18 mo control’ and ‘18 mo ΔTsc1’, the ‘12 mo control’ and ‘12 mo ΔRaptor’ in the third and fourth row in Fig. 3c should read ‘18 mo control’ and ‘18 mo ΔRaptor’, and the ‘si+Scn1a’ in Fig. 6i should read ‘si-Scn1a’. To correct these mistakes, we replaced the incorrect images and labels and rearranged the panels in Fig. 1a, Fig. 2c–e, Fig. 3b, c and Fig. 6i, k. Although this correction does not affect the results and conclusion of the paper, all the authors agree to correct these errors, and feel sorry and sincerely apologize for all the inconvenience.

The original article^1^ has been updated.
