# Supplementary material for: mTORC1 induces plasma membrane depolarization and promotes preosteoblast senescence by regulating the sodium channel Scn1a
Source: Bone Res. 2022 Mar 8;10:25. doi: 10.1038/s41413-022-00204-1 (PMC8901653; doi:10.1038/s41413-022-00204-1)
Supplement: Supplementary file 1 — Supplemental Information [file 41413_2022_204_MOESM1_ESM.pdf]

mTORC1 induces plasma membrane depolarization and promotes preosteoblast senescence through regulating the sodium channel Scn1a

Ajuan Chen et al.

Inventory of Supplementary information

Supplementary Figures (Supplementary Figures 1-13)

Legends to Supplementary Figures

Supplementary Table (Supplementary Table 1)

Supplementary Figure 1

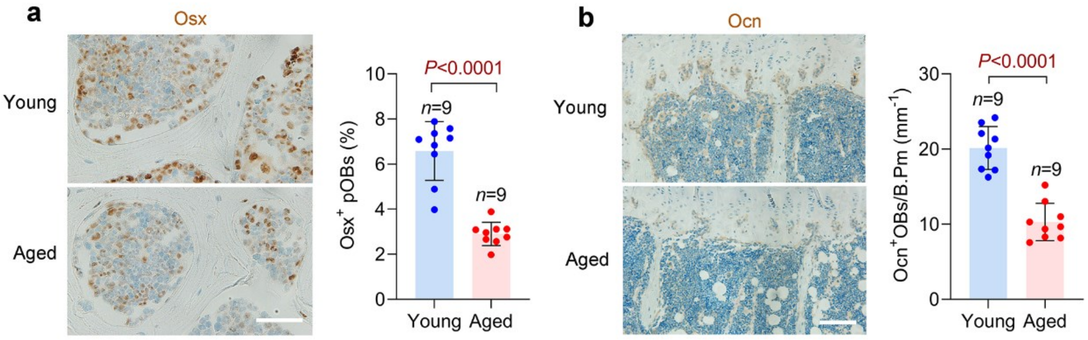

Supplementary Fig.1 Old mice present lower preosteoblast and osteoblast numbers than young mice. (a) Immunohistochemical (IHC) staining for osterix (Osx) in the tibias of young (9 month old) and aged (18 month old) C57 wildtype mice. Scale bar: 100 µm. Quantification of Osx-positive preosteoblasts (Osx^+ pOBs) relative to total cells (%) in the bone marrow is shown. **(b)** IHC staining for osteocalcin (Ocn) in tibias of the mice in a. Scale bar: 500 µm. Ocn-positive mature osteoblasts (Ocn^+ OBs) on the bone surface were measured as cells per millimeter of perimeter in sections (/B.Pm) (mm^{-1}). Data are shown as mean ± SD. The numbers of samples (n) are indicated in each figure panel. *P* values were determined by two-tailed Student's t test for single comparisons.

Supplementary Figure 2

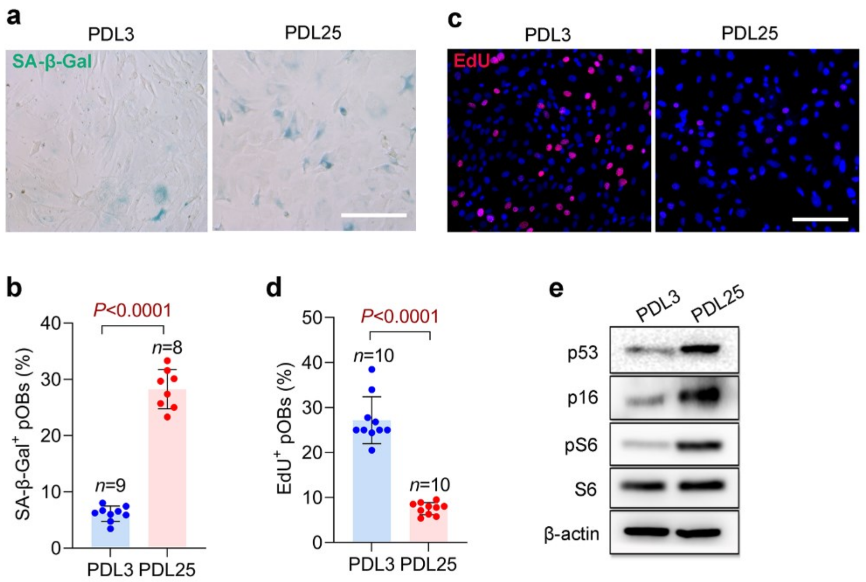

Supplementary Fig. 2 mTORC1 is activated in replicative senescent osteoblasts. (**a**) Primary calvarial osteoblasts at population doubling level (PDL) 3 and 25 were stained with SA-β-gal. Scale bar, 100 μm. (**b**) Quantification of SA-β-gal positive osteoblasts relative to total osteoblasts. (**c**) Immunostaining of EdU (red) in the cells and (**d**) quantitative analysis of EdU^+ cells relative to total cells. Scale bar, 100 μm in c. (**e**) Western blot analysis of senescent marker expression (p53 and p16) and mTORC1 activity (pS6(Ser235/236)) in the cells. Data are shown as mean ± SD. The numbers of samples (n) are indicated in each figure panel. *P* values were determined by two-tailed Student's t test for single comparisons.

Supplementary Figure 3

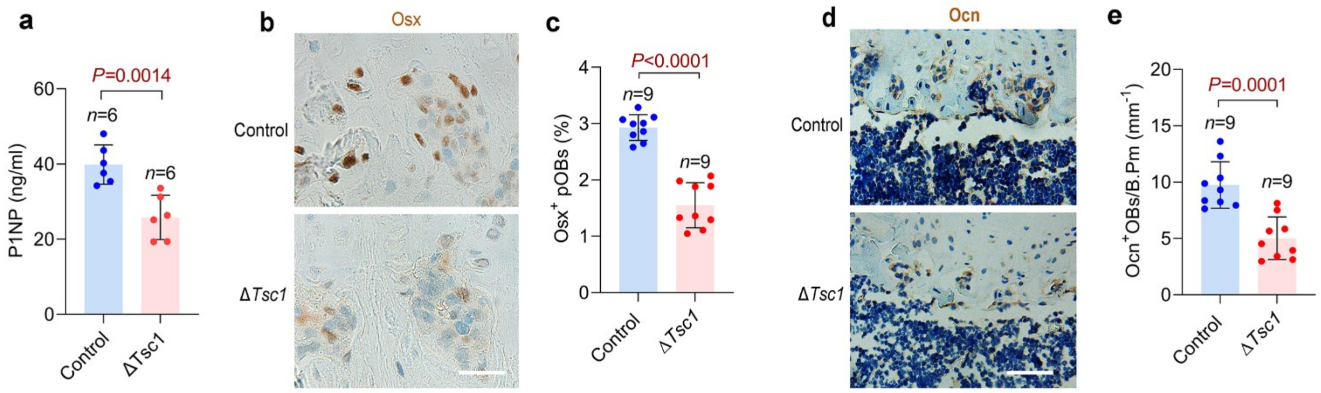

Supplementary Fig. 3 mTORC1 activation in preosteoblast results in reduced osteoblast number and activity in old mice. (a) Serum levels of P1NP of 18-month-old Δ*Tsc1* mice and their littermate controls determined by ELISA analysis. (**b**) IHC staining for Osx in the tibias of 18-month-old Δ*Tsc1* mice and their littermate controls. Scale bar: 50 μm. **(c)** Quantification of Osx-positive preosteoblasts (Osx^+ pOBs) relative to total cells in the bone marrow. **(d)** IHC staining for Ocn in the tibias of 18-month-old Δ*Tsc1* mice and controls. Scale bar: 100 μm. **(e)** Ocn-positive mature osteoblasts (Ocn^+OBs) on the bone surface were measured as cells per millimeter of perimeter in sections (/B.Pm). Data are shown as mean ± SD. The numbers of samples (n) are indicated in each figure panel. *P* values were determined by two-tailed Student's t test for single comparisons.

Supplementary Figure 4

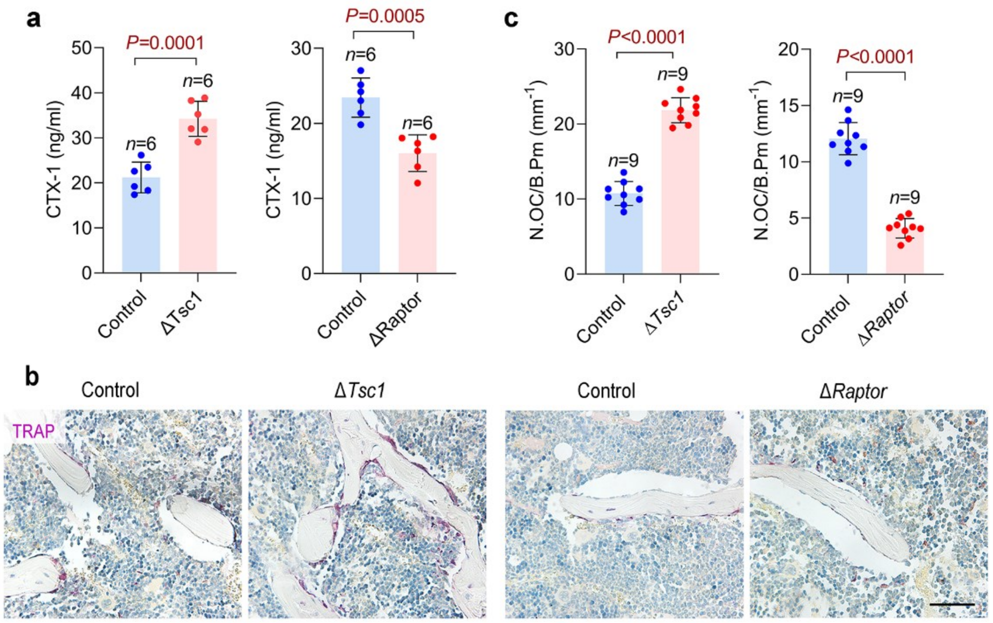

Supplementary Fig. 4 Hyperactive preosteoblastic mTORC1 increases, but inactive mTORC1 decreases, osteoclast formation and activity. (**a**) Serum levels of CTX-1 in 18-month-old Δ*Tsc1*, Δ*Raptor* and their control mice determined by ELISA analysis. (**b**) TRAP staining of distal femurs from 18-month-old Δ*Tsc1*, Δ*Raptor* and their control mice. Scale bar, 200 µm. (**c**) The number of osteoclasts (N.OC) on the bone surface was measured as cells per millimeter of perimeter in sections (/B.Pm). Data are shown as mean ± SD. The numbers of samples (n) are indicated in each figure panel. P values were determined by two-tailed Student's t test for single comparisons.

Supplementary Figure 5

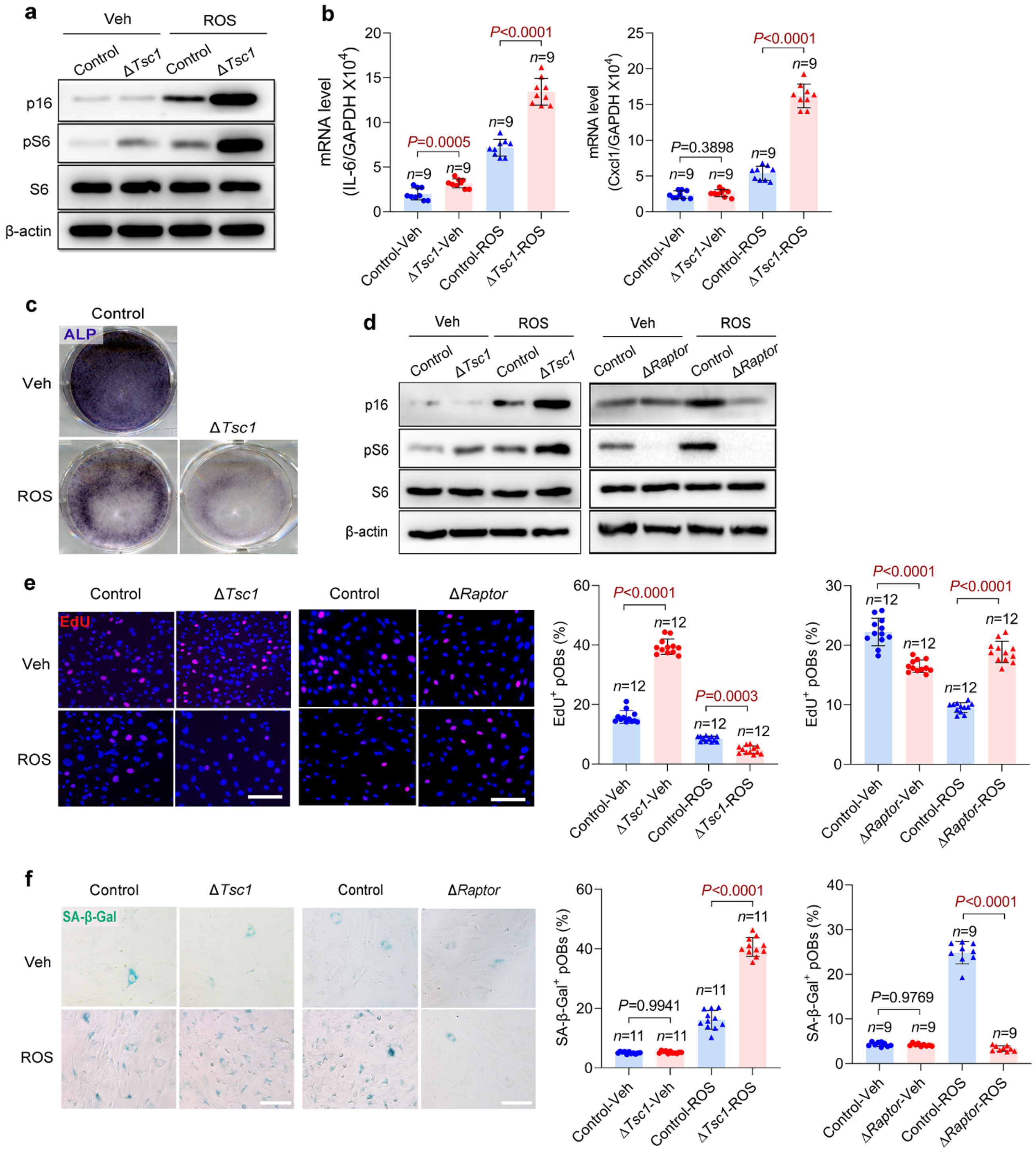

Supplementary Fig. 5 Hyperactive mTORC1 accelerates, but inactive mTORC1 prevents, preosteoblast senescence *in vitro*. (a) In primary calvarial osteoblasts

isolated from neonates Δ*Tsc1* and *Tsc1*$^{fl/fl}$ mice, senescence was induced by ROS. After 3 days, the cells were analyzed for senescence marker (p16 and p53) expression and mTORC1 activity (pS6) with western blotting. **(b)** qPCR analysis of IL-6 and Cxcl1 mRNA in the primary osteoblasts in a. **(c)** The cells were then induced to undergo osteogenic differentiation, and subjected to ALP staining on day 7 after differentiation induction. Primary osteoblasts isolated from long bone of Δ*Tsc1* and Δ*Raptor* mice were subjected to senescence induction by ROS. The cells were then subjected to analysis for p16 and pS6 expression (**d**), EdU immunostaining and quantitative analysis of EdU^{+} cells (**e**), and SA-β-gal staining and quantification of SA-β-gal^{+} cells (**f**). Scale bar, 100 μm in e. Data are shown as mean ± SD. The numbers of samples (n) are indicated in each figure panel. *P* values were determined by two-tailed Student's t test for single comparisons.

Supplementary Figure 6

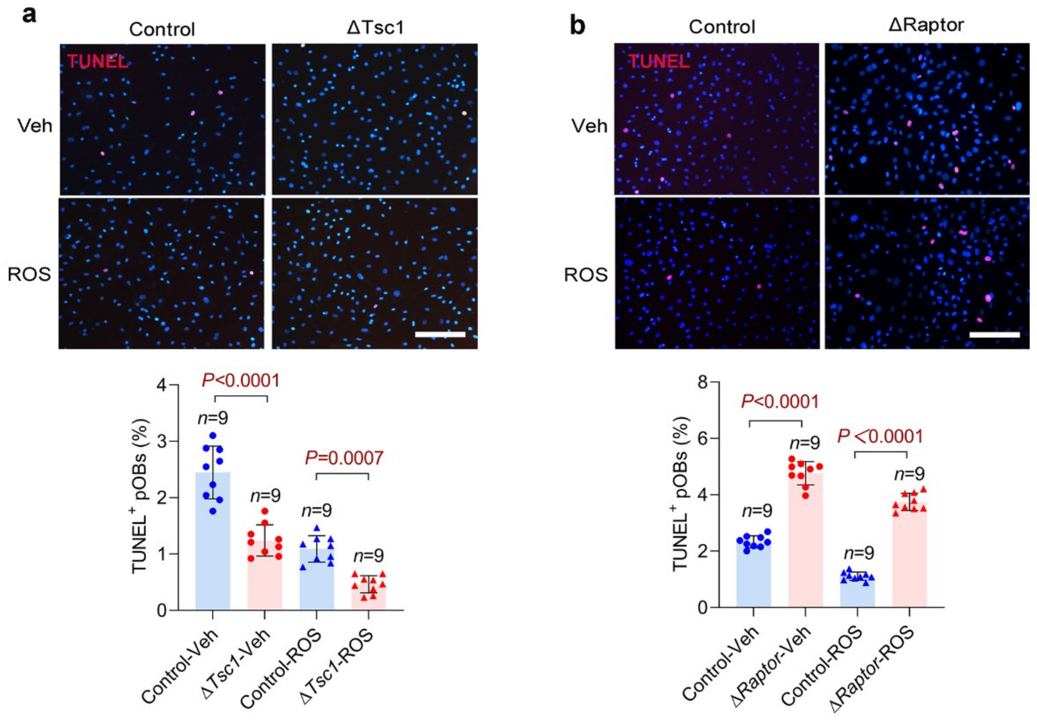

Supplementary Fig. 6 mTORC1 exerts consistent effects on apoptosis in replicative and senescent preosteoblasts. In primary calvarial osteoblasts isolated from neonates (a) Δ*Tsc1* and (b) Δ*Raptor* mice, senescence was induced by ROS. The cells were then subjected to TUNEL staining and quantification of $TUNEL^+$ cells. Data are shown as mean ± SD. The numbers of samples (n) are indicated in each figure panel. *P* values were determined by two-tailed Student's t test for single comparisons.

Supplementary Figure 7

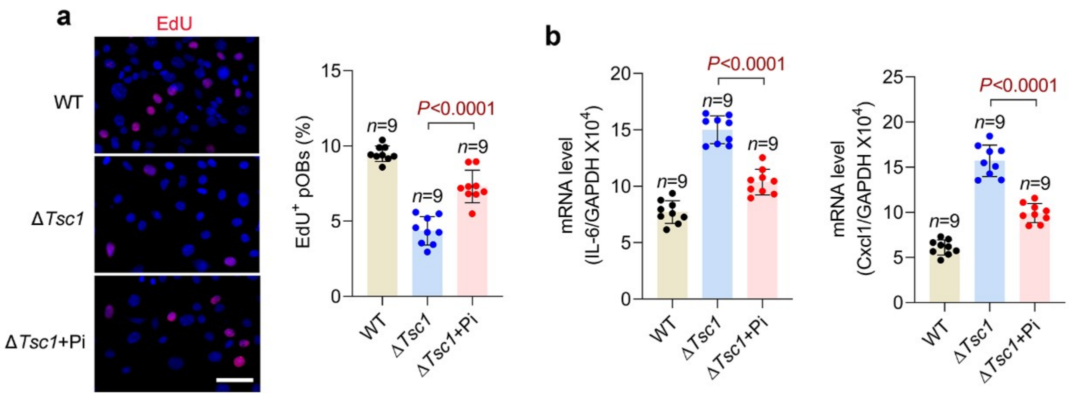

Supplementary Fig. 7 Pinacidil prevents depolarization and alleviates senescence in Δ*Tsc1* preosteoblasts. (a) Immunostaining of EdU in senescent Δ*Tsc1* preosteoblasts treated with pinacidil (Pi) and quantitative analysis of EdU^+ cells relative to total cells. Scale bar, 50 µm. **(b)** qPCR analysis of Cxcl1 and IL-6 mRNA in cells in a. Data are shown as mean ± SD. The numbers of samples (n) are indicated in each figure panel. *P* values were determined by two-tailed Student's t test for single comparisons.

Supplementary Figure 8

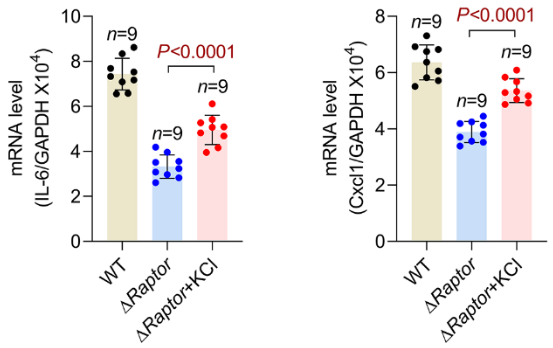

Supplementary Fig. 8 qPCR analysis of IL-6 and Cxcl1 mRNA in senescent Δ*Raptor* preosteoblasts treated with KCl. Data are shown as mean ± SD. The numbers of samples (n) are indicated. *P* values were determined by two-tailed Student's t test for single comparisons.

Supplementary Figure 9

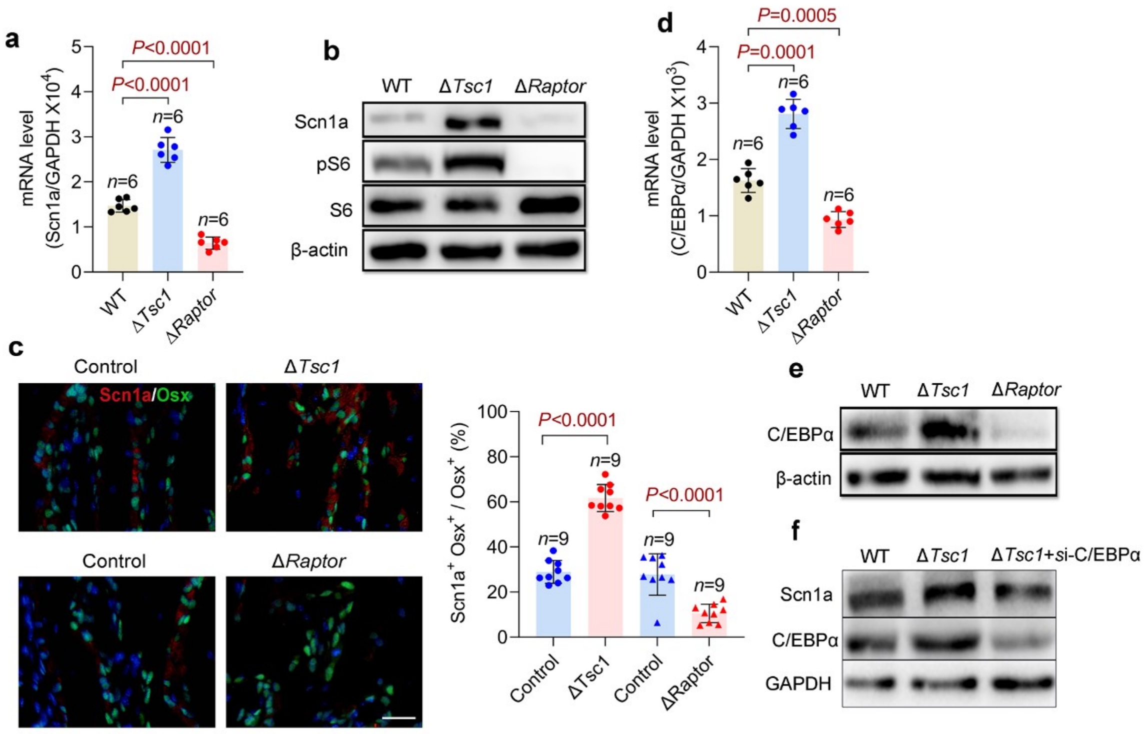

Supplementary Fig. 9 mTORC1 positively regulated Scn1a via C/EBPα. (a) qPCR and (**b**) western blot analysis of Scn1a expression in Δ*Tsc1* and Δ*Raptor* preosteoblasts. **(c)** Immunostaining of Scn1a and Osx in Δ*Tsc1* and Δ*Raptor* mice bone. Scale bar, 50 μm. Quantitative analysis of Scn1a$^+$ preosteoblasts (Scn1a$^+$ Osx$^+$) relative to total Osx$^+$ preosteoblasts. **(d)** qPCR and **(e)** western blot analysis of C/EBPα expression in Δ*Tsc1* and Δ*Raptor* preosteoblasts. **(f)** Δ*Tsc1* osteoblasts were subjected to C/EBPα knockdown with siRNA and subjected to western blot analysis of Scn1a expression. Data are shown as mean ± SD. The numbers of samples (n) are indicated. *P* values were determined by two-tailed Student's t test for single comparisons.

Supplementary Figure 10

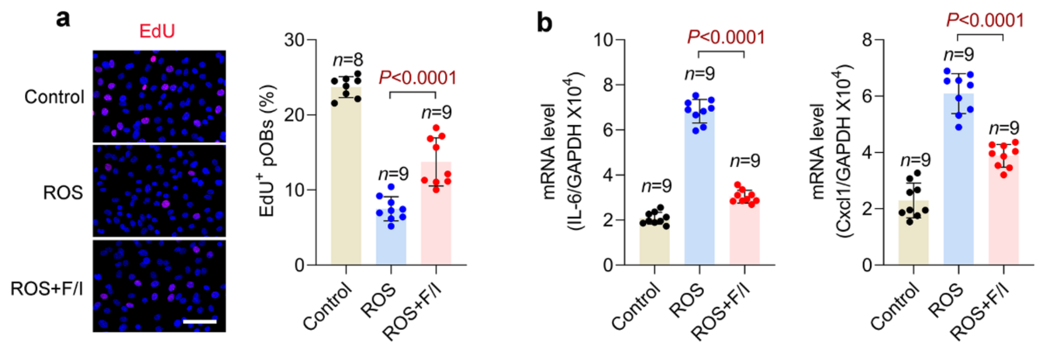

Supplementary Fig. 10 PKA activator abolished the senescent phenotype of preosteoblast induced by ROS. (a) ROS-induced senescent wildtype preosteoblasts were treated with F/I (forskolin + IBMx, PKA activator) or left untreated. The cells were immunostained with EdU and analyzed for of EdU^+ cells relative to total cells. Scale bar, 100 µm. **(b)** qPCR analysis of IL-6 and Cxcl1 mRNA in cells in a. Data are shown as mean ± SD. The numbers of samples (n) are indicated in each figure panel. *P* values were determined by two-tailed Student's t test for single comparisons.

Supplementary Figure 11

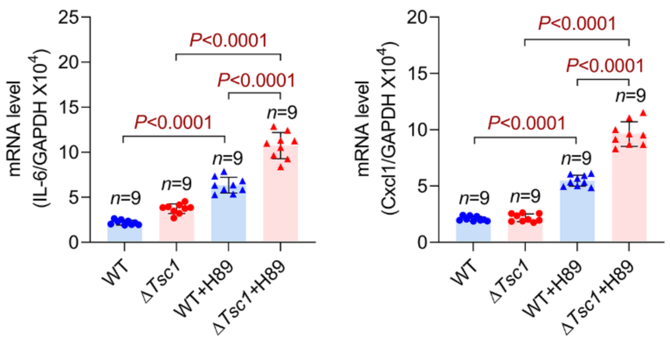

Supplementary Fig. 11 qPCR analysis of IL-6 and Cxcl1 mRNA in replicative WT and Δ*Tsc1* osteoblasts treated with H-89 (PKA inhibitor). Data are shown as mean ± SD. The numbers of samples (n) are indicated. *P* values were determined by two-tailed Student's t test for single comparisons.

Supplementary Figure 12

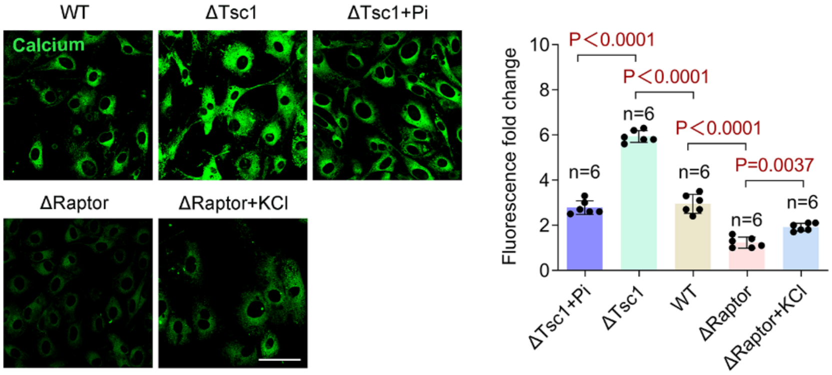

Supplementary Fig. 12 Cytosolic calcium levels change with the membrane potential in preosteoblasts obtained from long bones. Δ*Tsc1* and Δ*Raptor* preosteoblasts obtained from long bones were subjected to ROS induced senescence, treated with pinacidil (Pi) or KCl, and incubated with the Fluoforte probe to measure the relative cytosolic calcium levels. Calcium imaging data were quantified by normalizing the values to those of senescent wildtype (WT) preosteoblasts. Scale bar, 50 μm. Data are shown as mean ± SD. The numbers of samples (n) are indicated. *P* values were determined by two-tailed Student's t test for single comparisons.

Supplementary Figure 13 Uncropped versions of the gel images.

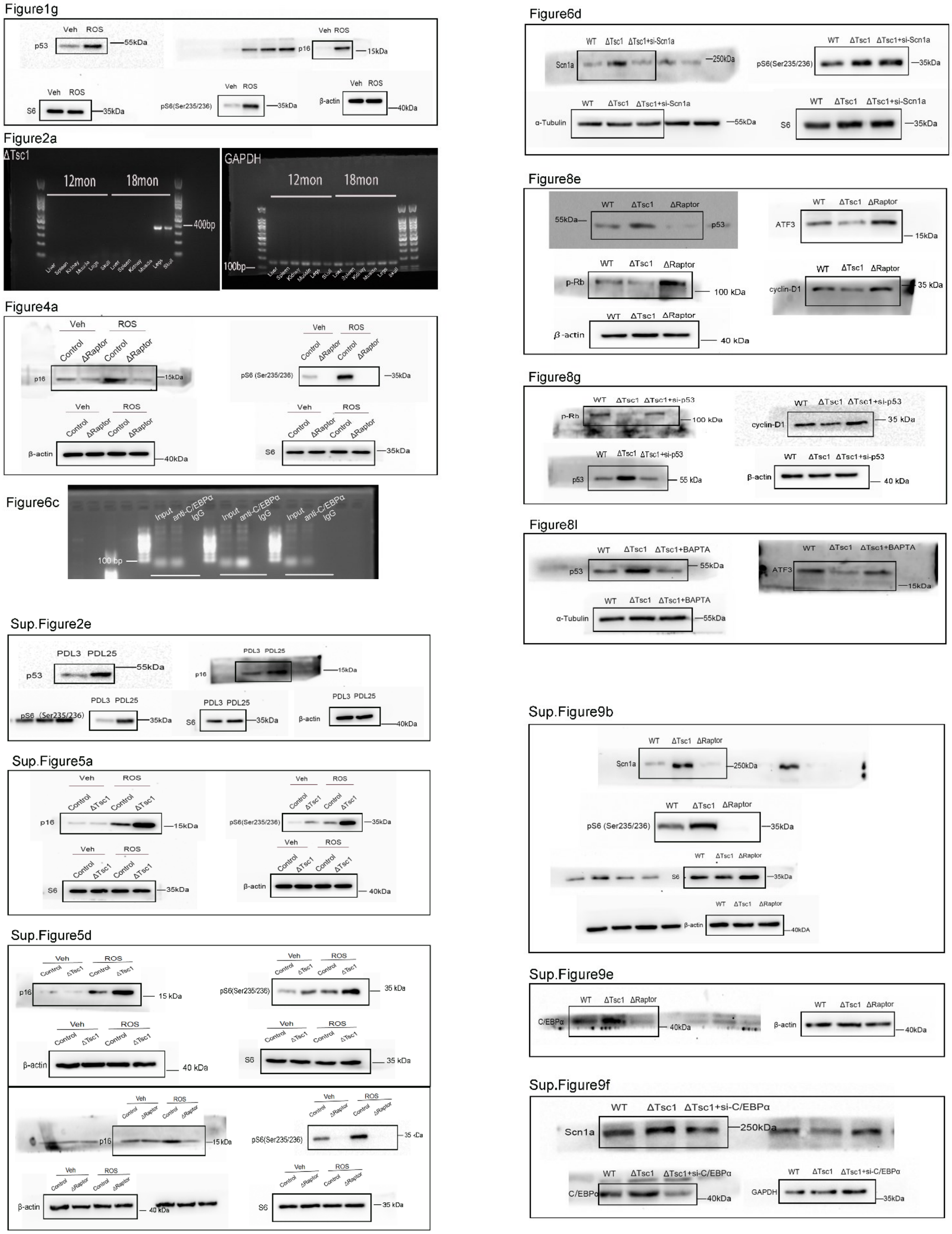

Supplementary Table

Supplementary Table 1. Nucleotide sequences of primers used for PCR

Gene	Strand	Sequence (5′ to 3′)
Osx-Cre	Forward	TACCAGAAGCGACCACTTGAGC
	Reverse	GCACACAGACAGGAGCATCTTC
Tsc1$^{flox/flox}$	Forward	GTCACGACCGTAGGAGAAGC
	Reverse	GAATCAACCCCACAGAGCAT
Recombined *Tsc1*	Forward	AGGAGGCCTCTTCTGCTACC
	Reverse	TGGGTCCTGACCTATCTCCTA
Raptor $^{flox/flox}$	Forward	CTCAGTAGTGGTATGTGCTCAG
	Reverse	GGGTACAGTATGTCAGCACAG
C/EBPα	Forward	TGGACAAGAACAGCAACGAG
	Reverse	TCACTGGTCAACTCCAGCAC
IL-6	Forward	GCTACCAAACTGGATATAATCAGGA
	Reverse	CCAGGTAGCTATGGTACTCCAGAA
Cxcl1	Forward	CTGGGATTCACCTCAAGAACATC
	Reverse	CAGGGTCAAGGCAAGCCTC
Scn1a	Forward	AGCCTATCCCTCGACCTGGA
	Reverse	CTGGTCATCCGTTTCCACCA
Scn1a 5′-UE	Forward	TGCTTGCTGCTGCCAATACT
	Reverse	GTTTCCACAGGCCGGTAGTG
GAPDH	Forward	GCACAGTCAAGGCCGAGAAT
	Reverse	GCCTTCTCCATGGTGGTGAA
